# A highly immunogenic vaccine platform against encapsulated pathogens using chimeric probiotic *Escherichia coli* membrane vesicles

**DOI:** 10.1038/s41541-022-00572-z

**Published:** 2022-11-26

**Authors:** Ryoma Nakao, Hirotaka Kobayashi, Yusuke Iwabuchi, Kazuyoshi Kawahara, Satoru Hirayama, Madeleine Ramstedt, Yuki Sasaki, Michiyo Kataoka, Yukihiro Akeda, Makoto Ohnishi

**Affiliations:** 1 Department of Bacteriology I, National Institute of Infectious Diseases, 1-23-1, Toyama, Shinjuku-ku, Tokyo, 162-8640 Japan; 2 Department of Pathology, National Institute of Infectious Diseases, 1-23-1, Toyama, Shinjuku-ku, Tokyo, 162-8640 Japan; 3 Department of Pediatric Dentistry/Special Needs Dentistry, Graduate School of Medical and Dental Sciences, Tokyo Medical and Dental University, 1-5-45, Yushima, Bunkyo-ku, Tokyo, 113-8519 Japan; 4 College of Science and Engineering, Kanto Gakuin University, 1-50-1, Mutsuura-higashi, Kanazawa-ku, Yokohama, Kanagawa 236-8501 Japan; 5 Division of Microbiology and Infectious Diseases, Niigata University Graduate School of Medical and Dental Sciences, 2-5274, Gakkocho-dori, Chuo-ku, Niigata, 951-8514 Japan; 6 Department of Chemistry, Umeå Centre for Microbial Research (UCMR), Umeå University, SE-901 87 Umeå, Sweden; 7 Nanostructures Research Laboratory, Japan Fine Ceramics Center, 2-4-1 Mutsuno, Atsuta-ku, Nagoya, 456-8587 Japan; 8 National Institute of Infectious Diseases, 1-23-1, Toyama, Shinjuku-ku, Tokyo, 162-8640 Japan

**Keywords:** Conjugate vaccines, Preclinical research, Conjugate vaccines, Conjugate vaccines

## Abstract

Vaccines against infectious diseases should elicit potent and long-lasting immunity, ideally even in those with age-related decline in immune response. Here we report a rational polysaccharide vaccine platform using probiotic *Escherichia coli*-derived membrane vesicles (MVs). First, we constructed a probiotic *E. coli* clone harboring the genetic locus responsible for biogenesis of serotype 14 pneumococcal capsular polysaccharides (CPS14) as a model antigen. CPS14 was found to be polymerized and mainly localized on the outer membrane of the *E. coli* cells. The glycine-induced MVs displayed the exogenous CPS14 at high density on the outermost surface, on which the CPS14 moiety was covalently tethered to a lipid A-core oligosaccharide anchor. In in vivo immunization experiments, CPS14^+^MVs, but not a mixture of free CPS14 and empty MVs, strongly elicited IgG class-switch recombination with a Th1/Th2-balanced IgG subclass distribution without any adjuvant. In addition, CPS14^+^MVs were structurally stable with heat treatment and immunization with the heat-treated MVs-elicited CPS14-specific antibody responses in mouse serum to levels comparable to those of non-treated CPS14^+^MVs. Notably, the immunogenicity of CPS14^+^MVs was significantly stronger than those of two currently licensed vaccines against pneumococci. The CPS14^+^MV-elicited humoral immune responses persisted for 1 year in both blood and lung. Furthermore, the CPS14^+^MV vaccine was widely efficacious in mice of different ages. Even in aged mice, vaccination resulted in robust production of CPS14-specific IgG that bound to the pneumococcal cell surface. Taken together, the present probiotic *E. coli* MVs-based vaccine platform offers a promising, generalizable solution against encapsulated pathogens.

## Introduction

Bacteria produce structurally stable nanoparticles called membrane vesicles (MVs) that are shed into the extracellular milieu. MVs have versatile functions in bacteria–host interactions by serving as vehicles carrying toxins, enzymes, immunomodulatory molecules and a wide range of signals for communications^1–4^. MVs contain both their outermost surface antigens and pathogen-associated molecular patterns (PAMPs), such as lipopolysaccharide (LPS), as well as lipoprotein, peptidoglycan, flagella, and DNA/RNA. Therefore, MVs function as acellular nanoparticle vaccines that protect against the pathogens from which they are derived. In clinical settings, MVs of group B meningococci have been used not only as a vaccine against invasive meningococcal disease since the 1980s^5^, but, very recently, also as a promising adjuvant against SARS-CoV-2^6^.

On the other hand, new approaches to produce semisynthetic MVs through innovative bioengineering technologies are also emerging. Generalized modules for membrane antigen (GMMA) technology for *Salmonella* MV vaccines is a representative technology that could be employed to deliver *Salmonella* lipopolysaccharide O-antigen to the immune system^7^. Irene et al. recently reported chimeric MVs expressing exogenous proteins were engineered with by using a lipoprotein transport pathway^8^. Exogenous polysaccharides were also introduced into *E. coli* MVs by tethering to the integral outer membrane protein PgaA^9^. Price et al. previously studied glycoengineered MVs made from an *E. coli* laboratory strain as a pioneering study, and reported that serum killing activity in mice intraperitoneally vaccinated with the MVs was comparable to that in mice vaccinated with PCV13^10^. However, so far, no genetically modified MV vaccine has been used in humans due to the lack of obvious advantages over currently licensed vaccines in the contexts of both safety and immunomodulatory capacity.

*E. coli* Nissle 1917 (EcN) is a probiotic strain commonly used in humans for improvement of gastrointestinal tract symptoms^11,12^. The EcN MVs showed an immunomodulatory effect on mammalian cells via TLR2^13^ and NOD1^14^. Rosenthal et al. reported that EcN MVs carrying a foreign protein antigen elicited Th1-biased immune responses, and proposed the applicability of EcN MVs as a vaccine vehicle for protein antigens^15^. On the other hand, the biotechnological breakthrough of detoxification of LPS-laden MVs and improvement of MV productivity are important matters to be addressed for the practical applications of MV vaccines in humans. We have recently reported a simple and efficient method using glycine to produce MVs that significantly reduced the amount of LPS from a flagella-deficient EcN derivative^16,17^. The bioengineered MVs was a potent adjuvant for the protein antigen in vivo despite of a dramatical decrease in the endotoxin content^16,17^. However, it remains to be investigated whether the detoxified EcN MVs have adjuvanticity to elicit immune responses of free polysaccharide antigen, and whether EcN MVs carrying exogenous polysaccharide antigen are immunogenic.

*Streptococcus pneumoniae* (pneumococcus) is one of major respiratory tract diseases worldwide. The capsular polysaccharide (CPS), of which >100 serotypes exist^18,19^, is one of its the most important virulence factors, as well as a promising vaccine target. Until now, pneumococcal vaccines have been developed as free CPS and protein carrier-conjugated CPS, which are currently licensed as the 23-valent pneumococcal polysaccharide vaccine (PPSV23) and the 10-valent or 13-valent pneumococcal conjugate vaccine (PCV10, PCV13), respectively. In response to other emerging serotypes beyond PCV13 due to the natural competence phenotype of this microorganism^20^, new PCV vaccines with broaden serotype, such as PCV15 or PCV20, are being tested in a range of clinical trials globally^21^, and have been already approved for use in adults in a few countries^22,23^. However, as the polysaccharides are per se T-cell independent antigen, insufficient immunogenicity not only of free purified CPS, but also of protein carrier-conjugated CPS has been described^24^. In addition, the manufacturing processes of both vaccines are complicated due to the need for CPS to be purified from the large-scale culture of the pathogens at a biosafety level 2 (BSL2) facility. Therefore, those vaccine products are expensive, and the cost-effectiveness is still controversial^25^.

In the present study, the glycine-induced probiotic *E. coli* MVs that display pneumococcal CPS at high density on their outermost surface were bioengineered and characterized to assess the applicability of this novel platform for vaccine development. Our findings highlight that the probiotic *E. coli* MV-based CPS vaccine significantly outperformed the currently available pneumococcal vaccines with respect to immunological responses in an in vivo study. Furthermore, the clinical applicability of the probiotic MVs-based vaccine platform is discussed in terms of safety, immunogenicity, and its utility for those with dampened immune responses such as the elderly.

## Results

### Characterization of MVs released from EcNΔflhD cells in the presence of glycine

**Fig. 1:**
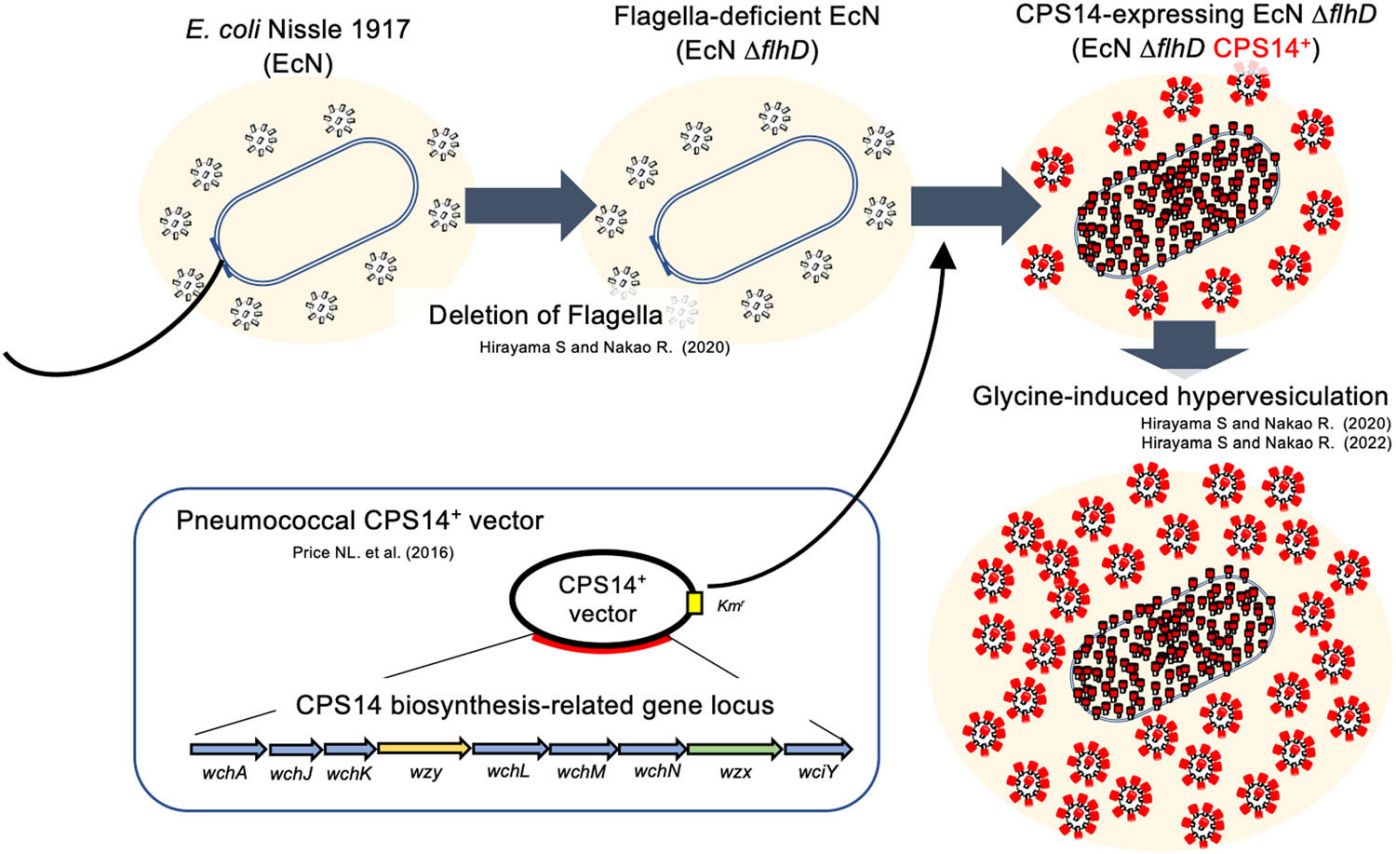
Schematic representation of the manufacturing process of probiotic *E. coli* MVs expressing pneumococcal CPS14. A large quantity of MVs with reduced endotoxin content was isolated from the supernatant of a flagella-deficient probiotic *E. coli* clone (EcN Δ*flhD*) cultured in LB broth supplemented with 1% glycine^16,17^. A pneumococcal CPS14 expression vector containing the entire CPS biosynthesis-related gene locus^10^ was introduced into the EcN Δ*flhD* strain. The resultant MVs expressed a chimeric LPS in which an *E. coli* O6 antigen repeating unit was replaced by a pneumococcal CPS composed of the serotype 14-specific tetra-saccharide repeating unit galactose-1-phophate-(β1-4)-N-acetylglucosamine-1-phosphate-(β1-3)-galactose-1-phophate-(β1-4)-glucose-^18^.

**Fig. 2:**
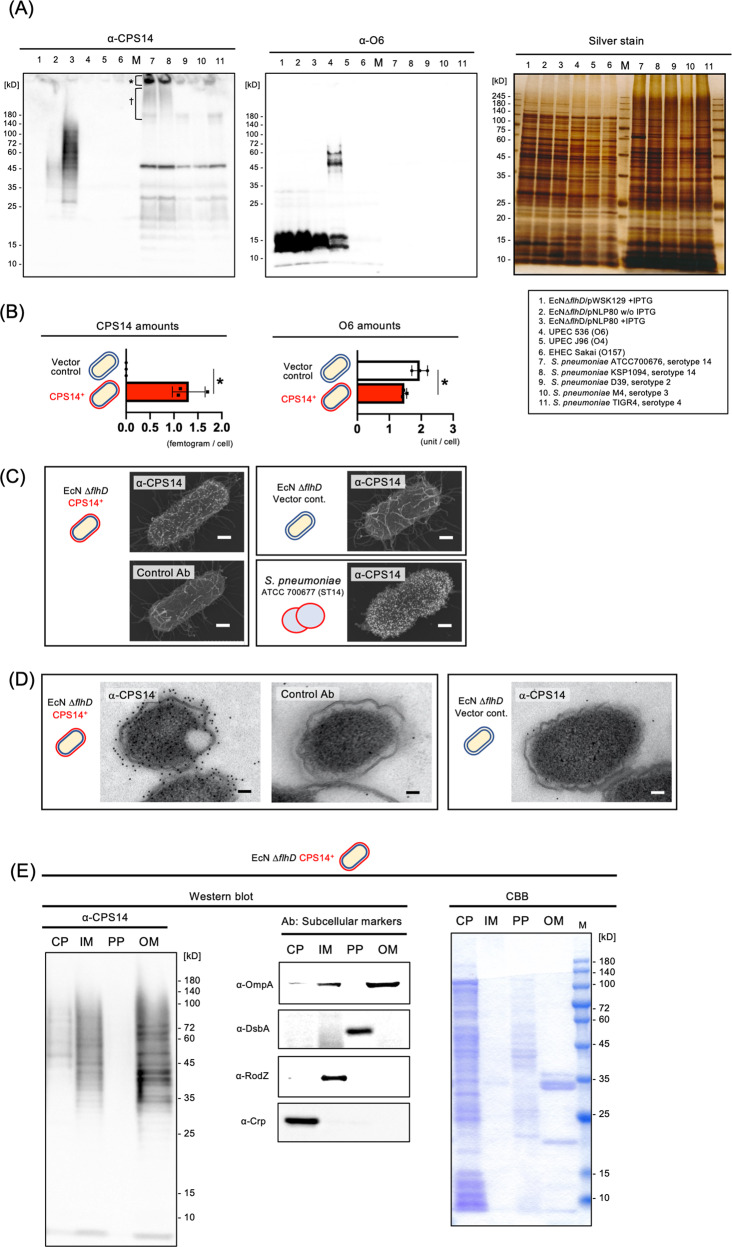
Pneumococcal CPS expression on EcN Δ*flhD* cells. **A** Western blot using anti-CPS14 (left panel) and anti-O6 antisera (middle panel), with sliver staining (right panel) as the loading control. The bacterial strains used in this experiment are shown with lane numbers at the lower right. For CPS14- and O6-probed western blot, the whole cells of all examined *E. coli* strains were standardized at an OD_600_ of 4.0, and the whole cells of all examined *S. pneumoniae* strains were standardized an OD_600_ of 8.0 using SDS-PAGE sample buffer. Twenty microliters of each sample was applied to the 12.5% polyacrylamide SDS-PAGE, electro-transferred onto a PVDF membrane, and probed with an anti-lipid A-core oligosaccharide antibody. For silver staining, 10-fold diluted whole-cell samples were prepared, *i.e*., whole cells of *E. coli* and *S. pneumoniae* were standardized an OD_600_ of 0.4 and 0.8, respectively, and twenty microliters of each sample was applied to the 12.5% polyacrylamide SDS-PAGE. Asterisk and dagger on CPS14-probed western blot membrane (left panel) denote CPS14-specific signals detected in the wells and the stacking gel of SDS-PAGE gel, respectively, shown in lanes 7 (*S. pneumoniae*, strain ATCC 700676 [serotype 14]) and 8 (*S. pneumoniae*, strain KSP1094 [serotype 14]). **B** Amounts of exogenous pneumococcal CPS14 (left panel) and *E. coli* original O6 (right panel) in whole cells of EcNΔ*flhD*/pWSK129 (vector control, *n* = 3) and EcNΔ*flhD*/pNLP80 (CPS14^+^, *n* = 3). All whole-cell samples used for analysis were collected from independent experiments. Data are expressed as the mean ± SD from results obtained in three independent experiments. *p ≤ 0.05, when performed with a Mann–Whitney *U*-test. **C** Immuno-FE-SEM. EcN Δ*flhD* cells harboring CPS14^+^ vector and the vector control, and *S. pneumoniae* strain ATCC 700676 (serotype 14) were probed with anti-CPS14 antibody (α-CPS14) or non-immunized rabbit serum (Control Ab). Primary antibodies were used at 1:500 dilutions. Anti-rabbit IgG antibody conjugated with 12-nm colloidal gold were used as the secondary antibody at 1:50 dilutions. Shown are representative images with scale bars indicated at the lower right. Bars: 200 nm. **D** Immuno-TEM. Whole cells of EcN Δ*flhD* harboring CPS14^+^ vector and the vector control were incubated with α-CPS14 or the control Ab. The primary antibodies were used at 1:500. Anti-rabbit IgG antibody conjugated with 12-nm colloidal gold was used as the secondary antibody at 1:50 dilutions. Shown are representative images with scale bars indicated at the lower right. Bars: 200 nm. **E** Subcellular fractionation of CPS14-expressing EcN Δ*flhD* cells. Subcellular fractions (cytoplasm [CP], inner membrane [IM], periplasm [PP], and outer membrane [OM]) were probed with α-CPS14, and with antisera against the following subcellular marker proteins: Crp [CP], RodZ [IM], DsbA [PP], and OmpA [OM]. Subcellular fractions were also stained with CBB. A 20-fold concentrated cytoplasmic fraction (CP), a 100-fold concentrated inner membrane fraction (IM), a 20-fold concentrated periplasmic fraction (PP), and a 100-fold concentrated outer membrane fraction (OM) were prepared from the bacterial suspensions standardized to OD_600_ = 2.0. Ten microliter of each sample was analyzed by SDS-PAGE.

Figure 1 shows a schematic of the method for mass producing EcNΔ*flhD* MVs expressing CPS14. As shown in Fig. 2A, exogenous CPS14 was found to be expressed as diffuse band signals in EcNΔ*flhD* cells harboring a pneumococcal CPS14 expression vector (pNLP80) by IPTG induction (Fig. 2A, lane 3). Similar signals appeared when detected with the anti-lipid A-core oligosacchaide antibody (Supplementary Fig. 1, lane 3). In the CPS14-probed western blot (Fig. 2A), strong signals were also detected in the wells (*) and sodium dodecyl-sulfate polyacrylamide gel electrophoresis (SDS-PAGE) stacking gel (^†^) of 2 different serotype 14 strains of *S. pneumoniae*, ATCC 700676 and KSP 1094 (lanes 7 and 8). CPS14-specific immunoreaction to CPS14-expressing EcNΔ*flhD* (EcNΔ*flhD*/pNLP80) cells was confirmed by the CPS14-probed dot-blot analysis, which also showed strong signals with both the serotype 14 strain (ATCC 700676) and purified CPS14, used as positive controls (Supplementary Fig. 2), demonstrating exogenous expression of pneumococcal CPS14 in EcNΔ*flhD* cells. In QDB analysis using purified CPS14 as a standard, a single cell of EcNΔ*flhD*/pNLP80 was found to contain 1.31 ± 0.175 fg of CPS14 (mean ± SD) (Fig. 2B). On the other hand, the amount of EcN-derived LPS (serotype O6), which appeared as a characteristic semi-rough type without long O-polysaccharides (Fig. 2A and Supplementary Fig. 3) as previously reported^16,26^, was significantly reduced in the CPS14-expressing EcNΔ*flhD*/pNLP80 cells as compared to the EcNΔ*flhD*/pWSK129 cells (vector control) (Fig. 2A, B, and Supplementary Fig. 3). In the sugar composition analysis of LPS purified from whole cells of the EcNΔ*flhD* strains, we observed an increase in galactose (Gal) to 129% and a reduction in mannose (Man) by 46% when CPS14 was exogenously expressed (Supplementary Table 1). The increase in Gal (Supplementary Table 1) was probably due to the partial replacement of O6-LPS by CPS14-LPS, because Gal is contained in the tetra-saccharide repeating unit of CPS14 (one Glu, one GlcNAc, and two Gal residues)^18^, but not in the pentasaccharide repeating unit of O6 (one Glc, one GlcNAc, one GalNAc, and two Man residues)^26^. On the contrary, the reduction in Man (Supplementary Table 1) was also probably due to the partial replacement of O6-LPS by CPS14-LPS, because Man is contained in O6 but not in CPS14^18,26^. Taken together, we suggest that exogenous CPS14 production in EcNΔ*flhD* cells interferes with the O6 production due to binding competition between CPS14 and O6 for the lipid A-core oligosaccharide moieties that serves as anchors of LPS tethered to the outer membrane. In addition, the subcellular localization of CPS14 was examined by immuno-FE-SEM (Fig. 2C), immuno-TEM (Fig. 2D) and subcellular fractionation-based assay (Fig. 2E), demonstraing that CPS14 is localized mainly at the outer membrane of EcNΔ*flhD* cells.

By using the glycine-induction method^16,17^, an abundance of MVs was isolated from EcNΔ*flhD*/pWSK129 (vector control) and EcNΔ*flhD*/pNLP80 (CPS14^+^). The amounts of glycine-induced MVs (vector control) and glycine-induced CPS14^+^MVs (protein equivalent, per one-liter culture supernatant, mean ± SD) were found to be 8.23 ± 3.46 and 24.3 ± 13.5 mg, respectively (Table 1). While the mechanism causing the higher yield in the glycine-induced CPS14^+^MVs as compared to the glycine-induced MVs (vector control) remains unclear, the yield of those MVs induced by glycine was ~20- and 60-fold higher, respectively, than that of the non-induced EcNΔ*flhD* strain without a vector (0.406 ± 0.0868 mg, *n* = 3) (Table 1). In nano-flow cytometry analysis, the approximate number (per ng of protein) and size were comparable between the glycine-induced MVs (vector control) and glycine-induced CPS14 ^+^ MVs at ~2 × 10^6^ particles/ng and 55–60 nm in diameter (Table 1 and Supplementary Fig. 4). Using limulus assay findings, the amount of LPS in glycine-induced CPS14^+^MVs was calculated to be 10.2 ± 6.82 (endotoxin units [EU] × 0^3^/ng) (Table 1), significantly less than the amount in non-induced MVs of EcNΔ*flhD* strain without a vector (32.6 ± 12.7 EU × 10^3^/ng) (Table 1), again demonstrating that the present glycine-induction method is useful for isolation of MVs with significantly reduced LPS amounts.

**Table 1:** Glycine-induced MVs (vector control) and CPS14^+^MVs.

Numerical information	MVs (vector control)	CPS14^+^MVs
Yield (mg, protein equivalent/1 L culture supernatant)*^1^	8.23 ± 3.48	24.34 ± 13.46
Number of MVs (particles × 10^6^/ng, protein equivalent)*^2^	2.11 ± 0.159	2.38 ± 0.867
Diameter (nm)*^2^	60.5 ± 0.635	63.5 ± 2.03
Lipid A activity, protein equivalent (EU × 10^3^/ng)*^3^	Not determined	10.2 ± 6.82
Pneumococcal CPS14 amount (ag/single particle)*^4^	0.00 ± 0.00	5.54 ± 2.56
*E. coli* O6 amount (unit [O6]/single particle) *^1^	0.196 ± 0.0372	0.0372 ± 0.0221

*1: Shown are results (mean ± SD) obtained from glycine-induced MVs (vector control, *n* = 3) and glycine-induced CPS14^+^MVs (*n* = 5). A statistically significant difference was detected between the groups (*p* < 0.05). The yields of both MVs after induction with glycine was ~20- and 60-fold, respectively, higher than that of the non-induced EcNΔ*flhD* strain without any vector (0.406 ± 0.0868 mg, protein equivalent, per one-liter culture supernatant, mean ± SD, *n* = 3).

*2: Shown are results (mean ± SD) obtained from glycine-induced MVs (vector control, *n* = 3) and glycine-induced CPS14^+^MVs (*n* = 5). There was no significant difference between the groups. Note: NanoFCM used in the present study is a state-of-the-art flow cytometry system for nanoparticles, though has a technical limitation making it unable to detect particles smaller than 40 nm in diameter. Therefore, the number of smaller particles is not included.

*3: Lipid A activity was determined as endotoxin unit (EU) using limulus assay with LPS of *E. coli* O111:B4 as the standard. Shown are results (mean ± SD) obtained from glycine-induced CPS14^+^MVs isolated from EcNΔ*flhD*/pNLP80 (*n* = 3). Activity of the glycine-induced MVs (vector control) was not examined. On the other hand, the activity level of glycine-induced CPS14^+^MVs was determined to be ~3-fold lower than that of non-induced MVs isolated from the EcNΔ*flhD* strain without a vector (32.58 ± 12.67 [x10^3^ EU/ng], *n* = 3). There was a significant difference between the groups (*P* < 0.05).

*4: Shown are results (mean ± SD) obtained from glycine-induced MVs (vector control, *n* = 3) and glycine-induced CPS14^+^MVs (*n* = 5).

**Fig. 3:**
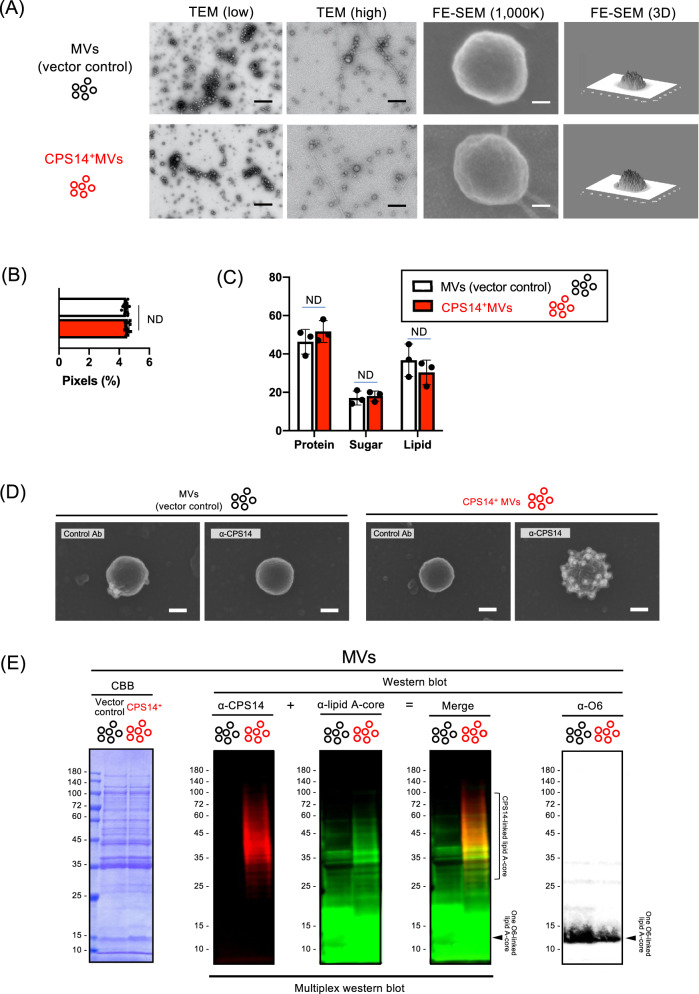

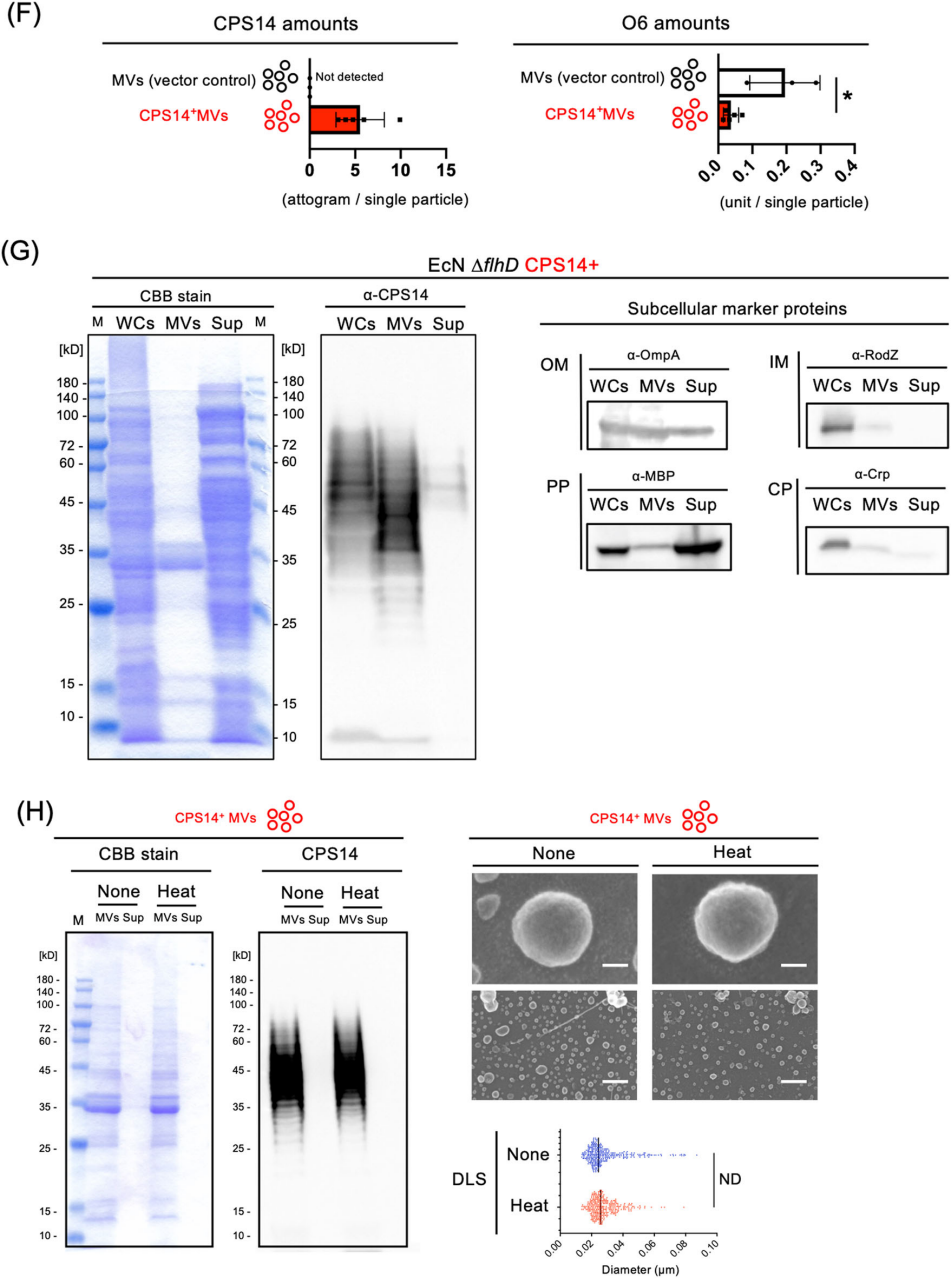
Characterization of CPS14^+^MVs. **A** Morphological analysis of MVs. Shown are representative images (TEM at low and high magnification, FE-SEM) with scale bars indicated at the lower right. Bars of TEM (low): 500 nm. Bars of TEM (high): 200 nm. Bars of FE-SEM (1000 K): 20 nm. FE-SEM 3D images are also shown at the right panel. **B** Surface roughness analysis of MVs by image-based profilometry. The surface roughness was quantified from five different areas (625 nm^2^) cropped from the center of both MVs (vector control) and CPS14^+^MVs (Supplementary Fig. 5). The results are expressed as the mean ± SD. ND: no statistically significant difference between MVs and CPS14^+^ MVs, when performed with a Mann–Whitney *U*-test. **C** MV surface compositions estimated from cryo-XPS data. Data are expressed as the mean ± SD from results obtained in three independent experiments. ND: no statistically significant difference between MVs and CPS14^+^ MVs, when performed with a Mann–Whitney *U*-test. **D** Immuno-FE-SEM of MVs. MVs (vector control) and CPS14^+^MVs were probed with α-CPS14 or the control Ab. The primary antibody was probed with anti-rabbit IgG antibody labeled with 12-nm colloidal gold. Shown are representative images with scale bars indicated at the lower right. Bars: 50 nm. **E** Multiplex western blotting of MVs (vector control) and CPS14^+^MVs for detection of both CPS14 and lipid A-core oligosaccharide on the same membrane. MV samples were prepared from EcNΔ*flhD*/pWSK129 (vector control) and EcNΔ*flhD*/pNLP80 (CPS14^+^) cells, and standardized at 4 µg/mL. Twenty microliters of each sample was applied to 12.5% polyacrylamide SDS-PAGE, electro-transferred to a PVDF membrane, and simultaneously probed with rabbit anti-CPS14 and mouse anti-lipid A-core oligosaccharide antibodies. Those primary antibodies were then simultaneously probed with SBB520-labeled anti-rabbit IgG and SBB700-labeled anti-mouse IgG as secondary antibodies, respectively. MV samples were also subjected to CBB staining as the loading control (left panel) and for O6-probed western blot (right panel). Square bracket at the right of the multiplex western blot membrane image denotes ladder signals corresponding to CPS14-linked lipid A-core oligosaccharides. Single bands of semi-rough type O6 LPS between 10 and 15 kDa are denoted by arrowheads at the right of the merged blot and O6-probed western blot membranes. **F** Amount of exogenous pneumococcal CPS14 (top panel) and *E. coli* original O6 (bottom panel) in MVs (vector control, *n* = 3) and CPS14^+^MVs (*n* = 5). Data are expressed as the mean ± SD from results obtained in 3 or 5 independent experiments. All MV samples used for analysis were collected from independent experiments. *p ≤ 0.05, when performed with a Mann–Whitney *U*-test. **G** MVs-tethered CPS14. Whole cells (WCs), MVs, and supernatants after ultracentrifugation (Sup) were prepared from the EcNΔ*flhD* cells expressing CPS14. Protein profiles and the presence of CPS14 were examined by CBB staining and Western blot probed with CPS14 antibody, respectively. The same sample set was also separately probed with antisera against the subcellular marker proteins Crp [CP], RodZ [IM], MBP [PP], and OmpA [OM]. The WC sample was standardized at 4.0 (OD_600_). The MV sample was standardized at 4 µg/mL. Using precipitation with trichloroacetic acid, the Sup sample had a 100-fold greater concentration than the original culture supernatant. Ten or twenty microliter of each sample was applied to 12.5% polyacrylamide SDS-PAGE. **H** Stability of CPS14^+^MVs after heat treatment (100 °C, 30 min). The total protein profiles of CPS14^+^MVs were examined following CBB staining (lower left panel) and CPS14-probed western blot. Appearance of CPS14^+^MVs without or with heat treatment are observed by FE-SEM. Bars in electron-micrographs at high magnification (top panels): 20 nm. Bars in electron-micrographs at low magnification (bottom panels): 200 nm. DLS results of MVs without (blue dots) and with heat treatment (red dots) are shown as scatter plots with vertical bars that denote the mean value. ND: no statistically significant difference, when performed with a Mann–Whitney *U*-test.

TEM and FE-SEM analysis showed that both MVs (vector control) and CPS14^+^MVs were spherical particles that range in size from 20 to 100 nm in diameter (Fig. 3A), which was in good agreement with the the nano-flow cytometry findings (Table 1 and Supplementary Fig. 4). Those preparations did not contain any flagella, which was confirmed by FE-SEM analysis (Fig. 3A) and flagellin-probed western blot analysis^16^. Although no difference was observed in the morphology (Fig. 3A), surface roughness (Fig. 3B and Supplementary Fig. 5) and relative composition of lipid, sugar, and protein (Fig. 3C and Supplementary Fig. 6), between MVs (vector control) and CPS14^+^MVs, CPS14 molecules were highly expressed on the outermost surface of CPS14^+^MVs, but not the vector control MVs (Fig. 3D, E). The amount of CPS14 in a single particle of CPS14^+^MVs was estimated to contain 5.54 ± 2.56 ag (mean ± SD, *n* = 3) (Fig. 3F), with a dramatic reduction in the amount of O6 in the CPS14^+^MVs as compared with the MVs (vector control), suggesting that CPS14 production interferes with the O6 production in MVs as well as whole cells. In addition, a multiplex western blottng, in which CPS14^+^MVs were probed with 2 antibodies against lipid A-core and CPS14, was performed (Fig. 3E). The findings confirmed that CPS14^+^MVs had a reduced amout of one O6-linked LPS and instead expressed polymerized CPS14 that exists as a lipid A-core oligosaccharide conjugate, i.e., a chimeric LPS.

In a comparative analysis of the constituents among whole cells, MVs, and the supernatant after ultracentrifugation (Fig. 3G), different than whole cells, MVs were found to contain abundant OmpA [OM], while MBP ([PP]), RodZ ([IM]), and Crp [CP] were only wealky detected. It was also confirmed that hypervesiculation following glycine addition was not the result of cell lysis, but rather “quasi-lysis” as previously reported^16^, because the supernatant contained a high level of MBP [PP], while Crp [CP] was undetectable (Fig. 3G). These findings suggest that glycine disrupted the integrity of the outer membrane, while that of the inner membrane was maintained.

### Comparison of humoral immune responses after immunization of CPS14^+^MVs and free CPS14 adjuvanted with empty MVs

**Fig. 4:**
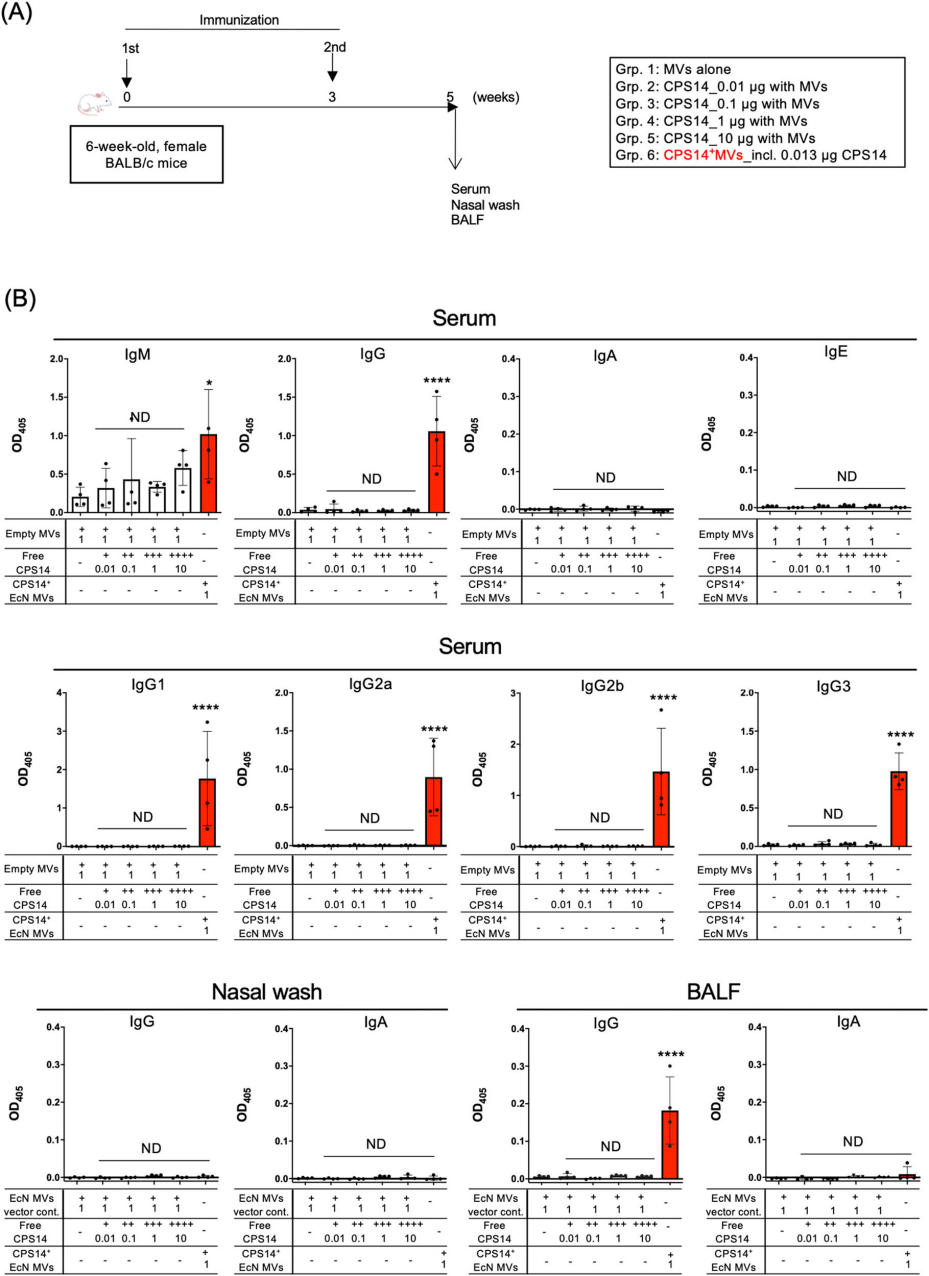
Comparison of humoral immune responses after vaccination with CPS14^+^ MVs or empty MVs mixed with different amounts of soluble CPS14. **A** Timeline of immunization. CPS14^+^MVs vs. soluble CPS14 antigens with CPS14-free MVs (Empty MVs). Six-week-old female BALB/c mice (*n* = 24) were subcutaneously immunized twice at weeks 0 and 3, with MVs alone, different amounts of CPS14 (0.01, 0.1, 1, and 10 µg) with empty MVs, and CPS14^+^MVs (*n* = 4, each). At 5 weeks, serum, nasal wash, and BALF samples were obtained. CPS14-specific antibody responses were analyzed by ELISA in which wells were coated with purified CPS14. **B** Serum IgM, IgG, IgA, and IgE (top panels) serum IgG subclasses (IgG1, IgG2a, IgG2b, IgG3) (middle panels), and IgG and IgA in nasal wash and BALF (bottom panels) were examined. In all ELISAs except serum IgG, samples were used at 1:100 dilution. Serum samples for IgG detection were used at 1:1000 dilution. The results are expressed as OD_405_ values (mean ± SD) after a 30-min incubation with AP substrate. ND: no statistically significant difference when compared to Grp. 1 (PBS). *p ≤ 0.05 when compared to Grp. 1 (PBS). ****p ≤ 0.0001 when compared to Grp. 1 (PBS). One-way ANOVA followed by Tukey’s multiple comparison test was used for the statistical analysis in Fig. 4.

The effectiveness of CPS14^+^MVs as a nanoparticle-based polysaccharide vaccine on acquired immunity was compared with that of free, nonparticulate CPS14 in an in vivo immunization model (Fig. 4A). In advance, the suitable dose of CPS14^+^MVs to elicit maximal immune responses was determined as 1 µg protein per a mouse by a preliminary experiment in which CPS14^+^MVs with different doses were injected in mice (Supplementary Fig. 7). Potent IgG responses were observed in both serum and BALF samples of CPS14^+^MVs group (Fig. 4B). In addition, we found a balanced IgG subclass distribution in the group of CPS14^+^MVs (Fig. 4B). In contrast, free, nonparticulate CPS14 elicited a limited immune responses with only weak IgM production (Fig. 4B). Neither IgA nor IgE were detected in any group (Fig. 4B). We found that remarkable IgG responses were induced by formalin-treated MVs, but not by MVs destroyed by SDS (Supplementary Fig. 8), which may indicate the importance of the structural integrity of MVs for immunogenicity. In addition, the thermostability of CPS14^+^MVs was examined (Fig. 3H) and those results showed that the total protein profiles, CPS14 expression/polymerization, morphology of the heat-treated CPS14^+^MVs were very similar to those without that treatment (Fig. 3H). Of interest, CPS14 was not detected in the supernatant after ultracentrifugation even after heat treatment, suggesting that the exogenous polymerized CPS14 molecules were not released into the supernatant but rather remained covalently bound to the lipid A core-oligosaccharide anchor. Furthermore, heat-treated CPS14^+^MVs induced a humoral immune response in mice that was as strong as that without heat treatment (Supplementary Fig. 8). Taken together, the results indicate that CPS14^+^MVs are highly thermostable.

### Long-term follow-up after administration with CPS14^+^MVs or two licensed pneumococcal vaccines, PPSV23 and PCV13

**Fig. 5:**
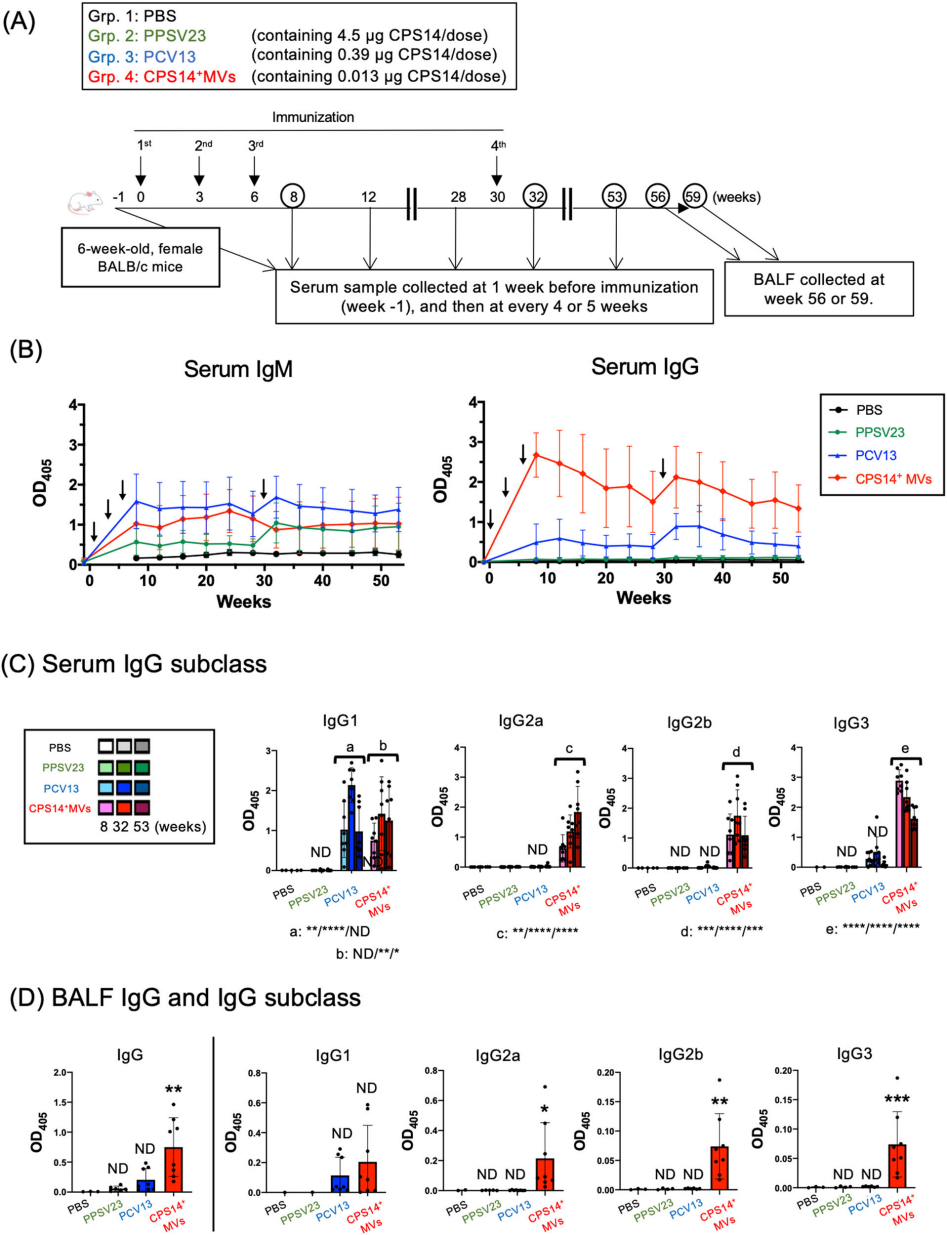

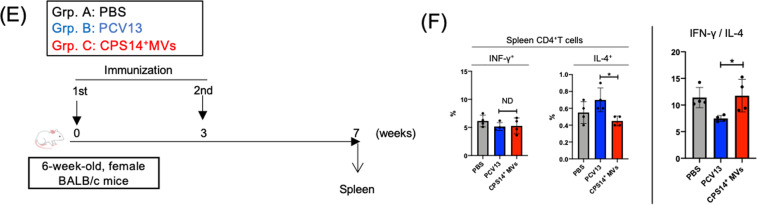
Long-term follow-up after vaccination with CPS14^+^ MVs or licensed vaccines. **A** Timeline of immunization: CPS14^+^ MVs vs. two currently available pneumococcal vaccines, PPSV23 and PCV13. Six-week-old female BALB/c mice (*n* = 27) were subcutaneously immunized with PBS, PPSV23, PCV13, or CPS14^+^MVs at weeks 0, 3, and 6 (PBS: *n* = 3, PPSV23: *n* = 8, PCV13: *n* = 8, CPS14^+^ MVs: *n* = 8). In addition, a booster (4th immunization) was administered at week 30. Serum samples were collected every 4 or 5 weeks starting from week 8. Pre-immune serum samples were also obtained 1 week before first immunization (week-1). At week 56 or 59, all mice were euthanized, and nasal wash and BALF samples were collected. Among the 27 mice used in this experiment, only two mice in the PPSV23 group died accidentally at 45 and 56 weeks, respectively. **B** Transition of CPS14-specific serum IgM and IgG. The OD_405_ values (mean ± SD) at each time point are connected by a colored line presenting each group. Arrows indicate the timing of immunization. **C** CPS-specific IgG subclass responses in serum at different time points (at weeks 8, 32, and 53). Serum IgG subclasses (IgG1, IgG2a, IgG2b, IgG3) were examined. Data are expressed as the mean ± SD. ND: no statistically significant difference. *p ≤ 0.05. **p ≤ 0.01. ***p ≤ 0.001. ****p ≤ 0.0001. Shown are the results of statistical analysis using one-way ANOVA followed by Tukey’s multiple comparison test, when compared to Grp. 1 (PBS) of the same age. **D** CPS-specific IgG and IgG subclass responses in BALF at week 56 or 59. ND: no statistically significant difference. *p ≤ 0.05. **p ≤ 0.01. ***p ≤ 0.001. Shown are the results of statistical analysis using one-way ANOVA followed by Tukey’s multiple comparison test, when compared to Grp. 1 (PBS) of the same age. In all ELISA except serum IgG, samples were used at 1:100 dilution. In ELISA for serum IgG, the samples were used at 1:1000 dilution. The results are expressed as OD_405_ values (mean ± SD) after a 30-min incubation with AP substrate. **E** Timeline of immunization, T-cell response. CPS14^+^ MVs vs. PCV13. Six-week-old female BALB/c mice were subcutaneously immunized with PBS, PCV13, or CPS14^+^MVs at weeks 0 and 3 (*n* = 4 per group). Spleens were harvested at week 8, and isolated splenocytes were used for intracellular cytokine assays. **F** After stimulation of splenocytes with PMA and ionomycin in the presence of GolgiPlug for 6 h, intracellular IFN -γ and IL-4 in CD4^+^T cells were examined. (Top panels) The percentages of IFN-γ^+^IL-4^-^CD4^+^T cells/Total CD4^+^T cells (IFN-γ^-+^cells) and IFN-γ^-^IL-4^+^CD4^+^T cells /Total CD4^+^T cells (IL-4^+^cells) are indicated in the lower right and upper left of each quadrant of flow cytometry findings. (Bottom panels) The percentages of IFN- γ^-+^cells and IL-4^+^cells, and ratio of IL-4/IFN-γ were compared between groups using one-way ANOVA followed by Tukey’s multiple comparison test. Data are expressed as the mean ± SD of the values.

Next, we compared the intensity and persistence of humoral immune responses between the CPS14^+^MVs vaccine and two currently licensed pneumococcal vaccines, *i.e*., PPSV23 and PCV13 (Fig. 5A). Administration with only 1 µg of EcNΔ*flhD*-derived CPS14^+^MVs, which contains 0.013 µg as CPS14 equivalent, elicited robust immune responses (Supplementary Fig. 7). On the other hand, administration with PPSV23 or PCV13 that contain the same CPS14 amounts (0.013 µg) did not induce immune responses sufficiently (Supplementary Fig. 7). So, regarding PPSV23 and PCV13, mice were administered with 90 µL of undiluted PPSV23 (incl. 4.5 µg as CPS14 equivalent), and undiluted PCV13 (incl. 0.39 µg as CPS14 equivalent), as their respective doses induced immune responses at maximal antibody responses (Supplementary Fig. 7). In the long-term follow-up after immunization, no or very little IgG was detected in the PPSV23 group through the 1-year timeframe, although a weak IgM production was maintained (Fig. 5B). On the other hand, the PCV13 group represented the strongest IgM responses among the groups, however, only weak or moderate IgG responses were observed in the PCV13 group (Fig. 5B). The IgG subclass distribution in the PCV13 group showed a typical Th2-skewed immune responses, i.e., abundant IgG1 but no or very little IgG2a in blood and lung (Fig. 5C, D). On the other hand, CPS14^+^MVs-elicited responses that persisted with a remarkably high level of IgG for 1 year (Fig. 5B), with balanced IgG subclass distributions, in which all IgG subclasses were strongly elicited in both blood and lung, which was in striking contrast to the PCV13 group (Fig. 5C, D). Neither IgA nor IgE were detected in any group during the timeframe of this study. Furthermore, T-cell response in mice following vaccination with PCV13 or CPS14^+^MVs was investigated (Fig. 5E). At 3 weeks after vaccination, the spleen was excised and CD4^+^T cell response was examined (Fig. 5E). There was no statistically significant difference regarding IFN-γ^+^T-cell population between the PCV13 and CPS14^+^MV groups (Figs. 5F and S9). On the other hand, the CPS14^+^MVs group showed a significantly larger IL-4^+^CD4^+^T-cell population as compared to the PCV13 group (Figs. 5F and S9). The ratio of IFN-γ^+^CD4^+^T cell/IL-4^+^CD4^+^T cell in mice of the CPS14^+^MVs group was significantly higher than those of the PCV13 group (Figs. 5F and S9). Taken together with findings showing elicitation of both Th1- and Th2-associated IgG subclass responses (Fig. 5C, D), it is considered that CPS14^+^MVs mitigate the risk of vaccine-induced immune enhancement by a Th1/Th2-balanced response, as compared to vaccination with PCV13, which shows a typical Th2-skewed IgG subclass response (Fig. 5C, D).

### CPS14^+^MVs enhanced potent humoral immune responses in mice of various ages

**Fig. 6:**
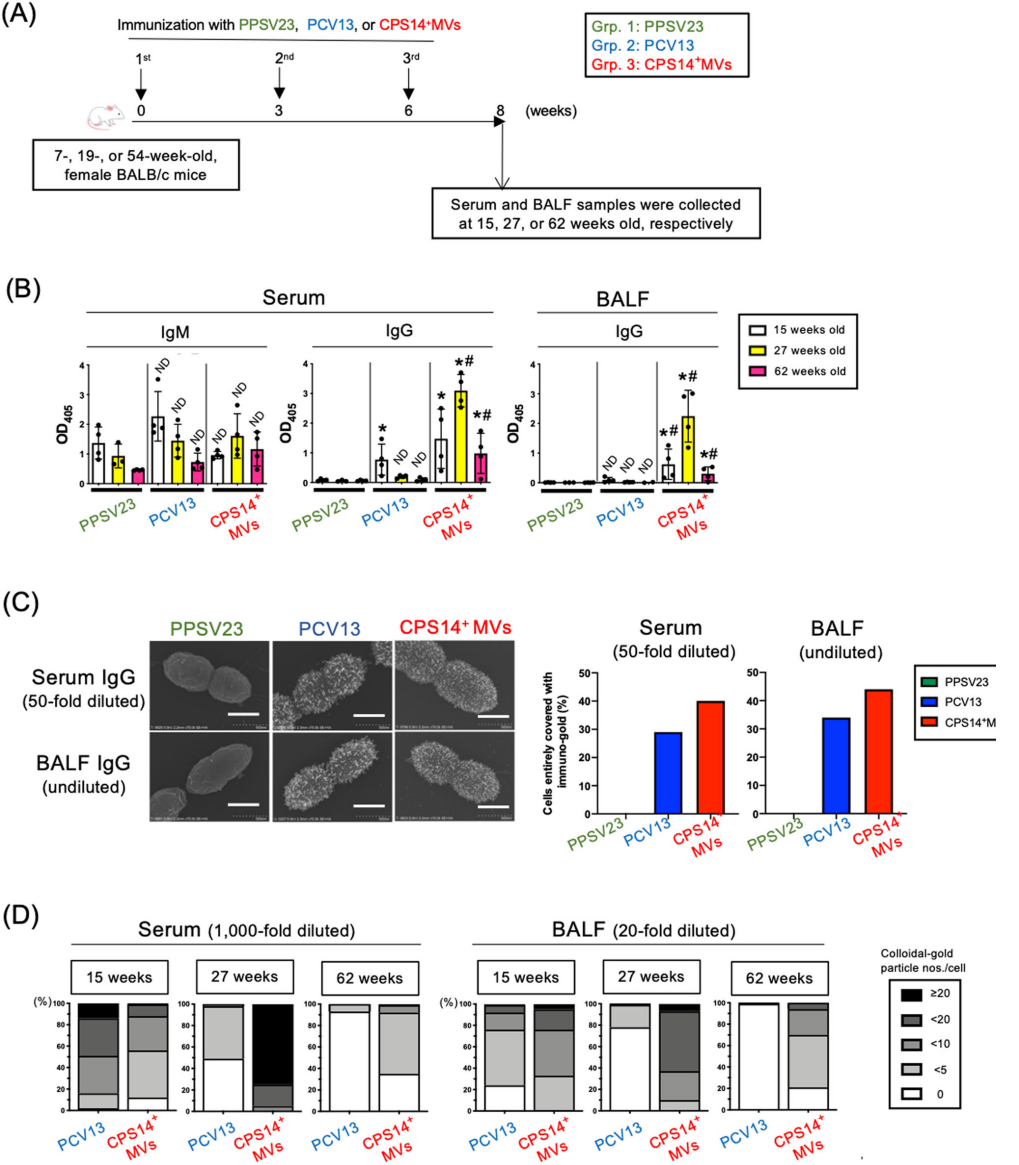
Immunization study using mice at different ages. **A** Timeline of immunization: CPS14^+^MVs vs. PPSV23 or PCV13 in mice at different ages. Thirty-six female BALB/c mice of different ages (7 weeks [*n* = 12], 19 weeks [*n* = 12], and 54 weeks [*n* = 12]) were subcutaneously immunized with PPSV23, PCV13, or CPS14^+^ MVs (PPSV23: *n* = 4 at each age, PCV13: *n* = 4 at each age, CPS14^+^ MVs: *n* = 4 at each age). **B** Humoral immune responses against CPS14. Serum IgM and IgG, and BALF IgG were examined. In ELISA for serum IgM and BALF IgG, the samples were used at 1:100 dilution. In ELISA for serum IgG, the samples were used at 1:1000 dilution. The results are expressed as OD_405_ values (mean ± SD) after a 30-min incubation with AP substrate. Shown are the results of statistical analysis using one-way ANOVA followed by Tukey’s multiple comparison test.ND: no statistically significant difference, when compared to Grp. 1 (PPSV23) of the same age. *p ≤ 0.05, when compared to Grp.1 (PPSV23) of the same age. ^**#**^*p* ≤ 0.05, when compared to Grp. 2 (PCV13) of the same age. **C** Reactivity of mouse serum and BALF to pneumococcal cells. After analyzing a serotype-14 laboratory strain, ATCC 700676, by immuno-FE-SEM, reactivities of serum and BALF samples with the cells were evaluated. The samples prepared from all the mice in each group (*n* = 4) at the same volume were used for the immuno-FE-SEM. The 50-fold diluted serum mixture and the undiluted BALF sample mixture of 15-week-old mice were used. Shown are representative the electron-micrographs, which are merge of the secondary electron image and the reflection electron image. Scale bars indicated at the lower right. Bars: 500 nm. In addition, 100 cells were also randomly selected in each group, and then the cells that were fully covered with immuno-gold were counted. The results of serum (50-fold diluted) and BALF (undiluted) are indicated by two bar graphs in the right. **D** The samples prepared from all the mice in each group (*n* = 4) at the same volume were used for the immuno-FE-SEM using a clinical isolate of serotype 14 pneumococcus obtained from patients with. The 1:1,000 diluted serum mixture and the 1:20 diluted BALF sample mixture of 15-, 27-, and 62-week-old mice were used. In total, FE-SEM images of 100 cells were randomly captured for each group at 5 × 10^5^-fold magnification. A rectangular area defined as 180 × 250 nm^2^ was cropped from the center of a pneumococcal cell. The number of immuno-gold particles on the surface per cell were counted.

To examine the effect of age on acquired immunity, three groups of mice of different ages were immunized with PPSV23, PCV13, and CPS14^+^MVs (Fig. 6A). Whereas the levels of serum IgM and IgG in the PPSV23 and PCV13 groups decreased with age, the mice in the CPS14^+^MVs group showed the highest antibody production at 27 weeks old (Fig. 6B). Among the three groups, only the mice in the CPS14^+^MVs group exhibited remarkable immune responses in both serum and BALF at all the ages (Fig. 6B). Notably, even 62-week-old mice immunized with CPS14^+^MVs could elicit robust CPS14-specific IgG responses in both serum and BALF, which was in contrast to the PPSV23 and PCV13 groups, which had no or little IgG production (Fig. 6B).

In the mice at 15 weeks old, the trend of IgG production observed in Fig. 6B was confirmed by immuno-FE-SEM analysis, which showed that mice in the groups of CPS14^+^MVs and PCV13 produced IgG antibodies in both 50-fold diluted serum and undiluted BALF that specifically bound to the outermost surface of live *S. pneumoniae* cells, while no or little IgG was detected in both serum and BALF of the PPSV23 group (Fig. 6C). Serum and BALF samples were further diluted 20-fold, and the diluted serum (1000-fold dilution) and BALF (20-fold dilution) samples were subjected to immuno-FE-SEM analysis again (Fig. 6D and Supplementary Fig. 10). In 15-week-old mice, the reactivity of the CPS14^+^MV group was comparable to that in the PCV13 group (Fig. 6D and Supplementary Fig. 10). On the other hand, in 27- or 62-week-old mice, CPS14^+^MVs vaccine was found to elicit functional IgG antibodies to *S. pneumoniae* at a higher level than PCV13 (Fig. 6D and Supplementary Fig. 10).

## Discussion

Considering the difficulty of inducing robust and long-lasting immune responses to polysaccharide antigens, the present study assessed the applicability of glycine-induced MVs as a novel vaccine platform against infection by encapsulated pathogens. EcNΔ*flhD* MVs were chosen as a vaccine carrier to investigate the prospective advantages in terms of: (1) safety based on the detoxified property with in vivo safety assessment^16^ and the practical use of live EcN cells in humans for more than 100 years^27^ and (2) the adjuvanticity of EcN MVs^16^ to be harnessed by a wide range of ligands of pattern recognition receptors^13–15^.

Our findings revealed that only 1 µg of EcNΔ*flhD*-derived CPS14^+^MVs (0.013 µg as CPS14 equivalent/µg of MVs) elicited robust T-cell responses and subsequent class-switch recombination to IgG, which was in sharp contrast to the response elicited by free CPS14 even when administered at high doses (~10 µg) with 1 µg of empty EcNΔ*flhD* MVs as an adjuvant (Fig. 4). Furthermore, CPS14^+^MVs strongly elicited IgG-dominant immune responses with balanced IgG subclass distributions, in which all IgG subclasses were strongly elicited in both blood and lung (Fig. 5).

Regarding PCV13, the antibody response profiles were IgM-dominant, and the total IgG level was significantly weaker as compared to the CPS14^+^MVs group (Fig. 5B). In addition, in the PCV13 group, the IgG subclass distribution was predominantly IgG1, while no or very little IgG2a was detected, suggesting that PCV13 is a typical Th2-type vaccine (Fig. 5C, D, F). The Th2-biased immunity is probably due to a Th2-enhancing adjuvant ingredient, aluminum phosphate^28^, which is supplemented in the PCV13 formula in order to make up for the weak immunogenicity of the conjugate vaccine.

Th1/Th2-balanced immune responses are essential for minimizing the risk of immediate hypersensitivity to vaccines, such as IgE-mediated anaphylaxis. In addition, robust IgG production with Th1/Th2-balanced immune responses is required for clearing pathogens, because both neutralizing antibody and cytotoxic T cells are important to induce protective immunity. Taken together, our in vivo studies highlight the lack of standard requirements of PCV13 for future vaccines, as well as the obvious advantage of the EcNΔ*flhD* CPS14^+^MV vaccine over two currently available pneumococcal vaccines.

A long-term follow-up study after immunization revealed that the CPS14^+^MV vaccine elicited strong IgG-dominant immune responses that were maintained for 1 year (Fig. 5). The finding indicates the persistence of long-lived antibody-secreting plasma cells and memory B cells at germinal centers during the timeframe of the study. We suggest that CPS14^+^MVs can be delivered to and remain at the local lymph nodes of the administration site, and subsequently interact with the immune cellular network, which can orchestrate T-cell-dependent IgG class switching and ensure protection against pneumococcal infection.

Successful vaccination of the elderly against pathogens that cause high morbidity and mortality represents a growing public health priority^29,30^. In particular, a reduction in the immune response to vaccines with aging is a critical issue to be addressed in light of our aging society. Thus, there is an urgent need to develop rational vaccines designed to elicit strong and persistent immune responses, even in low-responders such as infants and the elderly. Another in vivo immunization study with mice at different ages suggested that the CPS14^+^MVs vaccine was applicable for all age groups studied, i.e., 7-, 19-, and 54-week-old mice (Fig. 6). Considering the substantial decline in antibody responses in vaccinated 54-week-old mice in all the groups (Fig. 6), it is particularly meaningful that CPS14^+^MV-vaccinated, 54-weeks-old mice achieved robust IgG production in serum and BALF. This may be true that EcNΔ*flhD* MV-based vaccine system is applicable for subjects with dampened immune responses such as the elderly.

In contrast to the robust IgG production in mice of the CPS14^+^MVs group, IgA antibodies was not elicited by parenteral administration with CPS14^+^MVs, as well as PPSV23 or PCV13 (Fig. 4). Considering the importance in secretory IgA for protection at mucosal surfaces, mucosal administration may offer a more protective immunity than parenteral administration. Further studies are required such as in vivo protection assay by administration with CPS14 + MVs, both parenterally and intranasally.

The primary mechanism behind immunological advantages of glycine-induced EcN MVs over conventional vaccines remains obscure. A wide range of PAMPs in the EcN MVs and the ratio of PAMPs contents will be vital for the strong and Th1/Th2-balanced immune responses^13–15^. The structural integrity of MVs seemed to be vital for triggering the class-switch recombination from IgM to IgG (Supplementary Fig. 8). We also suggest that the surface pattern array of CPS14 antigens at high density on the MVs (Fig. 3) may ensure the favorable Th1/Th2-balanced immune responses after MV administration, because high-density antigens displayed on nanoparticle vaccines are known to enhance the antigen presentation ability of dendritic cells^31,32^, B-cell activation via crosslinking of B-cell receptors^33–35^, and balanced Th1/Th2 responses^36^. It also has been reported that nanoparticle vaccines ranging from 10 to 100 nm in diameter can drain from interstitial tissue into lymphatic vessels, and then accumulate within local lymph nodes^37^, which is in good agreement with the physical property of CPS14^+^MVs, which ranged in size from 20 to 100 nm in diameter (Table 1 and Fig. 3A, H), and the subsequent strong and persistent T-cell-dependent immune responses after CPS14^+^MV administration (Figs. 4, 5, and 6).

In a view of the significant cost of producing PCV13^24,38^, our probiotic MVs-based vaccine platform is able to significantly reduce the time and cost required for novel vaccine development by: (1) using an innovative, cost-effective glycine-induction method, (2) eliminating the need to manufacture at a BSL2 facility, and (3) enabling design flexibility based on a simple vector construction. In the future, the glycoengineered EcN Δ*flhD* MVs-based vaccine strategy can be applied for vaccine development to protect against not only other serotypes of pneumococci, but also other clinically important encapsulated pathogens, such as Group B streptococcus, *Neisseria meningitidis*, *Acinetobacter baumannii*, *Klebsiella pneumoniae*, among others.

Principle of glycoengineering of the chimeric EcNΔ*flhD* MVs is based on the ligation of exogenous polysaccharides from the pyrophosphate-undecaprenyl (PP-Und) anchor to the lipid A-core oligosaccharide anchor^10^, whose enzymatic reaction is catalyzed by the O-antigen ligase, WaaL^39–41^. Then, the transport of the lipid A-core polysaccharides takes place from the outer leaflet of the inner membrane to the outer leaflet of the outer membrane by the action called “PEZ” model^42^. In the present study, a pneumococcal PP-Und-linked CPS14 was cleaved from PP-Und, and subsequently linked to the lipid-A core block. The resultant chimeric LPS were translocated onto outer membrane of EcNΔ*flhD* strain (Fig. 2). The glycine-induced MVs also exogenously expressed polymerized CPS that existed as chimeric LPS on the surface (Fig. 3). Although WaaL is known to exhibit a highly relaxed specificity toward the lipid moiety of the donor substrate^43,44^, to what extent LPS biosynthesis system of EcNΔ*flhD* allow exogenous polysaccharides with different species and sizes remains to be elusive. For that reason, further functional studies of this platform applicability to various polysaccharides should be assessed with the better understandings of molecular mechanism of chimeric LPS biosynthesis and expression on MVs.

In conclusion, we succeeded in overproduction of probiotic EcNΔ*flhD* MVs with high-density CPS14 on the outermost surface (Fig. 3). In in vivo immunization experiments, CPS14^+^MVs strongly elicited IgG class-switch recombination with a Th1/Th2-balanced IgG subclass distribution without any additional adjuvant (Figs. 3, 4, 5). The CPS14 ^+^MVs-elicited humoral immune responses persisted for 1 year in both blood and lung (Fig. 5). Notably, the immunogenicity of CPS14^+^MVs was significantly stronger as compared to that of either of the two licensed pneumococcal vaccines (Figs. 5 and 6). Furthermore, the CPS14^+^MVs vaccine was found to be widely applicable for mice of different ages, because vaccination, even in aged animals, resulted in robust production of CPS14-specific IgG bound to the pneumococcal cell surface (Fig. 6). Taken together, we suggest that the probiotic *E. coli* MV vaccine platform can provide a generalizable solution for defense against capsulated pathogens.

## Methods

### Bacterial strains and growth conditions

A schematic overview of the bioengineering of probiotic *E. coli* cells expressing exogenous polysaccharides is shown (Fig. 1). In brief, a flagella-deficient clone of *E. coli* strain Nissle 1917 (EcNΔ*flhD*) was used as “a mini factory” for MV production^16,17^. EcNΔ*flhD* was further transformed by a low-copy plasmid pWSK129 (vector control)^45^ or the derivative plasmid pNLP80 which contains an entire locus responsible for pneumococcal CPS14 biogenesis^10^. Both pWSK129 and pNLP80 carry a kanamycin-resistance marker and an isopropyl-β-d(-)-thiogalactopyranoside (IPTG)-driven *lac* promoter. All *E. coli* strains were grown either in LB broth or on LB agar at 37 °C in aerobic conditions. Kanamycin sulfate (Fujifilm Wako Pure Chemical, Osaka, Japan) and IPTG (Fujifilm Wako Pure Chemical) were also supplemented at 50 µg/mL and 0.01 mM, respectively, when required. Two strains of *Streptococcus pneumoniae*, ATCC 700676 and KSP1094 were also used as representative serotype-14 strains of laboratory and clinical strains, respectively, to evaluate the reactivity of antibodies produced in immunized mice. Both strains were grown on brain heart infusion-based blood agar at 37 °C in an 5% CO_2_ incubator or in an anaerobic chamber (miniMACS, Don Whitley Scientific Ltd., Shipley, UK) in 80% N_2_, 10% H_2_, and 10% CO_2_.

### Preparation and chemical analysis of LPS

LPS was isolated from freeze-dried *E. coli* cells by a hot-phenol extraction method^46^, with some modifications. In brief, *E coli* cells were collected from 250-mL culture for 16 h at 37 °C in aerobic conditions, and freeze-dried. The freeze-dried cells suspended with 10 mL of distilled water was mixed with 10-mL of 90% phenol, and stirred for 30 min at 68 °C. The water-phase was collected after centrifugation at 12,000 × *g*, and dialyzed against distilled water for 3 days. The dialyzed LPS solution was freeze-dried (crude LPS). The crude LPS was washed once with distilled water by ultracentrifugation at 100,000 × *g* to prepare purified LPS for chemical analysis. Analysis of sugar and fatty acid composition was performed by gas-liquid chromatography (GC-1014s, Shimadzu, Kyoto, Japan)^47^.

### MV isolation

Bacterial culture supernatant was collected by centrifugation at 7190 × *g* for 30 min at 4 °C from 16-h culture of *E. coli* grown in LB broth supplemented with 1% glycine. The collected supernatant was filtered through a 0.22-µm-pore PVDF membrane to eliminate contaminated cells, and was subsequently ultracentrifuged at 100,000 × *g* for 2 h to yield MVs as sediments. Thanks to the peptidoglycan-weakened effect of glycine, supplementation with 1% glycine to LB broth resulted in an ~70-fold increase in the yield of MVs as assessed by protein amount when compared to MVs not induced with glycine^16,17^. On the other hand, the endotoxin activity of glycine-induced MVs was ~8-fold reduced compared to that of non-induced MVs^16,17^. The detoxified MVs were resuspended in 20 mM Tris-HCl (pH 8.0) and stored at −20 °C. The protein concentration of MVs was measured by Bradford assay^48^ using bovine serum albumin (BSA) as a standard.

### Thermostability of CPS14^+^MVs

CPS14^+^MVs standardized at a concentration of 1 mg/mL with PBS (pH 7.4) were treated without or with heating at 100 °C for 30 min. Following ultracentrifugation of both non- and heat-treated CPS14^+^MVs, the resultant sediment and supernatant after ultracentrifugation were subjected to SDS-PAGE analysis. The morphology of CPS14^+^MVs without or with heat treatment was examined by FE-SEM and a Zetasizer (Zetasizer Pro, Malvern, UK). MV samples without or with heat treatment were also used for in vivo immunization experiments. The timeline is shown in Fig. S8D.

### Surface roughness

MV surface topography was evaluated by an indirect, SEM micrograph-based profilometry by Gwyddion software ver. 2.58 as reported previously^49^ with some modifications. In brief, for analysis of MV surface morphology in detail, field emission scanning electron microscopy (FE-SEM) was operated at an accelerating voltage of 5 kV, a short working distance of 2.0- or 2.1-mm working distance, 10^6^-fold magnification. The edge detection method was performed by cropping the center areas (30 × 30 nm^2^) of MVs with the sizes of 60 ± 10 nm in diameter. Fifteen images were randomly selected and analyzed in both MVs (vector control) and CPS14^+^MVs, respectively. In the roughness detection, the different values from adjacent pixels were obtained regardless of background and curved surfaces like MVs. Since the zero-crossing often highlights noise, the processing includes smoothing by the Laplacian of Gaussian and noise reduction by setting thresholds as follows: the full width at half maximum of Gaussian is 10 pixels, and the threshold amount of normalized root-mean-square error is 1.0. The surface roughness of MVs was calculated as a percentage of outline/total number of pixels.

### Nano-flow cytometry (nFCM)

The size and concentration of MV samples were analyzed using a NanoFCM (NanoFCM Inc., Xiamen, China) according to the manufacturer’s instructions. Briefly, a cocktail of silica nanospheres (SiNPs) with four different diameters (68, 91, 113, and 155 nm) (NanoFCM Inc.) was used for calibration before nFCM analysis. PBS (buffer alone) was also analyzed as a background signal. MV concentration and size distribution were calculated using the nFCM software package, NanoFCM v. 2.0.

### Quantitative dot-blot (QDB) analysis of pneumococcal CPS14 and *E. coli* O6 polysaccharide antigens

The amounts of pneumococcal CPS14 and *E. coli* O6 polysaccharides in whole cells and MVs were quantified by quantitative dot-blot (QDB) analysis. Bacterial cells were heat inactivated at 100 °C for 15 min, standardized at OD_600_ = 2.0, and serially diluted 2-fold with PBS (pH 7.4). MVs were standardized at 100 ng/µL (calculated as protein equivalent) were serially diluted 2-fold with 20 mM Tris-HCl (pH 8.0). For QDB of pneumococcal CPS14, purified CPS14 was used as the standard (#76943, Statens Serum Institut [SSI], Hillerød, Denmark). The CPS14 prepared at the concentration of 10 ng/mL were 2-fold serially diluted. For QDB analysis of *E. coli* O6 polysaccharides, heat-inactivated whole cells of the *E. coli* O6 reference strain^50^ were used as the standard. Whole cells standardized at OD_600_ = 2.0 were serially diluted 2-fold with PBS (pH 7.4). The amount of O6 contained in 1 CFU of the O6 reference strain was defined as one unit of the O6 amount. The signal intensity of each sample was quantified based on standard curves for CPS14 and O6, which were generated based on the average of signal intensities obtained in duplicate assays of purified CPS14 and the *E. coli* O6 reference strain, respectively, on the same membrane. Ten microliter of each solution was blotted onto PVDF membrane, and evaporated at 50 °C for 5 min. The blotted membrane was blocked with 1% skim milk (SM) in PBS containing 0.05% Tween 20 (PBST) for 16 h at 4 °C or for 2 h at 37 °C, and then was incubated with anti-CPS14 antiserum (#16753, SSI) diluted at 1:10,000 dilution. Horseradish peroxidase (HRP)-labeled anti-rabbit IgG (GE Healthcare, Buckinghamshire, UK) was used as the secondary antibody at 1:200,000 dilution. Chemiluminescence after addition of HRP substrate with high sensitivity (Western BLoT Hyper HRP Substrate, Takara-bio, Shiga, Japan) or with ultra-high sensitivity (Immobilon ECL Ultra Western HRP Substrate, Merck, Darmstadt, Germany) was quantified by a densitometry program of Fusion solo (Vilber Lourmat, Marne-la-Vallée, France). Relative CPS14 amounts in MV samples were calculated based on the standard curve of CPS14 standards. To define the amount of CPS or O6 per cell, the number of EcNΔ*flhD* cells was estimated based on the turbidity of the bacterial culture after determining that the OD_600_ at 0.1 corresponded to 5 × 10^7^ CFU. To define the amount of CPS or O6 per single MV particle, the number of EcNΔ*flhD* MVs was estimated based on the total protein amounts of MVs, using data obtained from nano-flow cytometry (Table 1), in which one nanogram of total protein of the MVs (vector control) and CPS14^+^MVs corresponded to 2.11 × 10^6^ and 2.38 × 10^6^ particles (mean ± SD), respectively.

### Subcellular fractionation

Subcellular fractionation of *E. coli* cells was performed by a standard protocol of a different solubilization technique by using 2% *N*-lauryl sarcosyl^51^.

### Limulus assay

To quantify LPS, a limulus assay was performed using an Endospecy ES-50M kit (Seikagaku Co., Tokyo, Japan), according to the manufacturer’s instructions, with LPS from *Escherichia coli* O111:B4 (Sigma) as the standard.

### Cryo-X-ray photoelectron spectroscopy (Cryo-XPS)

The chemical surface composition of isolated MVs was analyzed by using a multivariate curve resolution analysis of carbon 1 s spectra obtained from Cryo-XPS analyses^52,53^. Briefly, cryo-XPS spectra were recorded from frozen suspensions of OMVs in PBS, at liquid nitrogen temperature (-160 °C). Thereafter, C 1 s spectra for each sample were fitted using Matlab (Mathworks Inc) with spectral envelopes previously determined and published representing the profiles for three major substance groups, *i.e*., peptides (proteins or peptidoglycan), lipids or polysaccharides. Using this methodology the content of C atoms from these three building blocks could be predicted at the outermost portion (less than ~10 nm depth) of the MV surface.

### SDS-PAGE and western blot

MV and whole-cell samples were separated by Tris-glycine SDS-PAGE using 12.5% polyacrylamide gels and stained with Coomassie brilliant blue (CBB). For Western blotting, gels were electroblotted onto PVDF membranes. Rabbit polyclonal antibodies against FliC flagellin^54^ were used at 1:2000 dilution. Rabbit polyclonal antibodies against CPS14 (#16753, SSI) were used at 1:10,000 dilution. Mouse monoclonal antibodies against MBP (E8032, New England Biolabs, Ipswich, UK) were used at a dilution of 1:10,000. Mouse monoclonal antibodies against lipid A-core oligosaccharide (Clone WN1 222-5, Hycult Biotech, Uden, Nederlands) was used at a dilution of 1:1000. Rabbit polyclonal antibodies against OmpA, DsbA, RodZ, and Crp were used as previously described^55^. HRP-labeled anti-rabbit IgG (GE Healthcare) was used as the secondary antibody at 1:200,000 dilution. Following addition of Western BLoT Hyper HRP Substrate (Takara-bio) or Immobilon ECL Ultra Western HRP Substrate (Merck, Darmstadt, Germany), chemiluminescence was visualized with Fusion solo (Vilber Lourmat). For detection of both lipid A-core oligosaccharide and CPS14 on the same membrane, fluorescence western blotting was performed using StarBright Blue (SBB) 520-labeled goat anti-rabbit IgG (Bio-Rad, Hercules, CA, USA) and SBB700-labeled goat anti-mouse IgG (Bio-Rad) as the secondary antibodies, in place of HRP-labeled antibodies. Fluorescence was visualized with Amersham ImageQuant 800 Fluor (Cytiva, Marlborough, MA, USA). Excitation was performed by use of a 460-nm blue light laser for both SBB520 and SBB700, and fluorescence emission was collected in the green range of the spectrum using a band pass filter of 525 ± 10 nm (emission maximum: 520 nm) for the SBB520 dye and in the infrared range of the spectrum using a band pass filter of 715 ± 15 nm (emission maximum: 700 nm) for the SBB700 dye.

### Electron and immuno-electron microscopy

Morphological analysis of MVs was performed by FE-SEM with sub-nanometer resolution (Regulus8220, Hitachi High-Tech, Tokyo, Japan)^16^, with modified protocol of sample preparation to be optimized for MVs. For FE-SEM analysis, MVs standardized at 100 ng/µL with 20 mM Tris-HCl (pH 8.0) were placed on poly-l-lysine-coated coverslips immobilized in 4-well multi-dishes (#176740, Thermo Fischer Scientific, San Jose, CA, USA) and incubated for 150 min at 15–25 °C to allow the MVs to attach on the coverslips. Subsequently, MVs were fixed with admixture of 2% paraformaldehyde and 2.5% glutaraldehyde for 16 h. The fixed samples were dehydrated in graded acetone solutions, and dried in a critical point dryer using CO_2_ (CPD 300, Leica Microsystems, Wetzlar, Germany). The samples were then coated with osmium vapor using an osmium plasma coater and finally visualized with FE-SEM (Regulus8220, Hitachi High-Tech, Tokyo, Japan). The 3D structure of MVs was constructed from FE-SEM images by ImageJ software (version 1.44, National Institutes of Health) equipped with interactive 3D Surface plot analysis at the following setting parameters: smoothing 2.0; perspective 0.0; lightning 0.2.

For immuno-FE-SEM, bacterial cells or MVs immobilized on the coverslips were incubated with 200 µL of 1% BSA in PBS for 30 min at 15–25 °C. After washing once with 0.1% BSA in PBS, samples were incubated with CPS14 antibody (SSI) or normal rabbit antibody in 0.1% BSA in PBS for 1 h at 15–25 °C. After washing three times with 0.1% BSA in PBS, samples were incubated with 200 µL of colloidal gold-labeled secondary antibody prepared with 0.1% BSA in PBS for 1 h at 15–25 °C. In the present study, goat anti-rabbit IgG (H + L) secondary antibody conjugated with 12-nm colloidal gold (#111-205-144, Jackson ImmunoResearch, West Grove, PA, USA) and goat anti-mouse IgG (H + L) secondary antibody conjugated with 10-nm colloidal gold (EMGMHL10, BBI solutions, UK) were used for probing primary rabbit and mouse antibodies, respectively. After immunoreaction procedures, fixation, dehydration, drying and osmium plasma coating were sequentially performed in the manner described in the previous paragraph.

For immuno-TEM of *E. coli* and *S. pneumoniae*, the cells were fixed with 4% paraformaldehyde for 2 h. After washing three times with 0.1 M phosphate buffer, the pellet of cells was embedded in agar, dehydrated with in a series of ethanol concentrations (50, 70, 80, 90, 95, and 100%), and embedded in LR white resin (Nisshin EM Co. Ltd., Tokyo, Japan). Ultrathin-sections on nickel grid were incubated with 1% BSA in PBS for 30 min at 15–25 °C. After washing once with 0.1% BSA in PBS, the sections were incubated with CPS14 antibody (SSI) or normal rabbit antibody in 0.1% BSA in PBS for 1 h at 37 °C. After washing three times with 0.1% BSA in PBS, the sections were incubated with 12 nm colloidal gold-labeled anti-rabbit IgG antibody (BBI solutions) 0.1% BSA in PBS for 1 h at 37 °C. After immunoreaction, the sections were stained with 4% uranyl acetate and lead citrate, and then analyzed with a transmission electron microscope (H-7700, Hitach High-Tech). For assessment of the immunoreactivity of the serum and BALF samples from mice, FE-SEM images of 100 cells were randomly captured for each group at 5 × 10^5^-fold magnification. A rectangular area defined as 180 × 250 nm^2^ was cropped from the center of a pneumococcal cell. The number of immuno-gold particles on the surface per cell was counted.

### Animal experiments

All animal experiments were approved by National Institute of Infectious Diseases (NIID) Institutional Animal Care and Use Committee (Protocol nos. 118149 and 121046) and performed in compliance with the commitee guidelines. In Figs. 4A, 5A, E, 6A, S7A, S8B, and S8D, the experimental overviews are shown with the timelines of immunization and euthanasia. Six-week-old female BALB/c mice (Japan SLC, Inc, Hamamatsu, Japan) were subcutaneously immunized in the back twice with a 3-week interval with isolated MVs at a dose equivalent of 1 µg of protein containing 0.013 µg of CPS14, CPS14 (SSI) at different doses [0.01, 0.1, 1, and 10 µg, CPS14 equivalent] or the combination of MVs and CPS14, for which the injection volume was 0.09 mL. In some experiments, 23-valent pneumococcal polysaccharide vaccines (PPSV23, pneumovax23, Merck) and 13-valent pneumococcal conjugate vaccines (PCV13, prevenar13, Pfizer, Rockville, MD, USA) were subcutaneously administered in the back of female BALB/c mice in the same manner. Different doses of PPSV23 (0.5, 1.5, 4.5 µg, CPS equivalent) and PCV13 (0.043, 0.13, 0.39 µg, CPS equivalent) were administered to the mice. In an experiment with a 1-year follow-up for persistence of antibody production, immunization with PBS, PPSV23 (4.5 µg, CPS equivalent), PCV13 (0.039 µg, CPS equivalent), and CPS14^+^MVs (0.013 µg, CPS equivalent) was performed for 4 times at the following ages of mice: 6, 9, 12, and 36 weeks. In another experiment, 7-, 19, and 54-week-old female BALB/c mice were subcutaneously immunized 3 times at 3-week intervals in the back with PPSV23 (4.5 µg, CPS equivalent), PCV13 (0.039 µg, CPS equivalent), and CPS14^+^MVs (0.013 µg, CPS equivalent). Serum and BALF samples were collected 2 weeks after the final immunization. Serum, nasal wash and bronchoalveolar lavage fluid (BALF) samples were collected from mice and used for detection of CPS14-specific antibodies by ELISA. In the procedure of antigen coating, CPS14 antigen was coated at 125 ng per well onto ELISA plates. Alkaline phosphatase (AP)-labeled anti-mouse IgG (H + L) was purchased from Thermo Fischer Scientific, and used at 1:1000 dilution. AP-labeled anti-mouse IgM, IgE, IgA, IgG1, IgG2a, IgG2b, IgG3 were purchased from Southern Biotech (Birmingham, AL, USA), and used at 1:1,000 dilutions. Chromogenic development using para-nitrophenyl phosphate were recorded at absorbance at 405 nm at 15, 30, 60, 120 min with the plate reader Cytation5 (Biotek, Winooski, VT, USA). For T-cell response study, BALB/c mice were subcutaneously immunized in the back twice with a 3-week interval with PBS, PCV13 (0.039 µg, CPS equivalent), and CPS14^+^MVs (0.013 µg, CPS equivalent). At 3 weeks after the final immunization, the mouse spleens were collected and analyzed.

### T-cell assessment using flow cytometry

Splenocyte suspensions were prepared by physically disrupting the spleen capsule and then passing the suspension through nylon mesh filter with a pore size of 70 µm. After removal of red blood cells by treatment with ACK lysis buffer, freshly isolated splenocytes standardized at 2 × 10^6^ cells in RPMI1640 with 10% FBS were stimulated with 5 µL of a cell activation cocktail with brefeldin A (#423303, Biolegend, San Diego, CA, USA) in a CO_2_ incubator for 6 h. After harvesting stimulated splenocytes, surface staining was performed with the following panel of antibodies and reagents: PerCP anti-mouse CD3ε (clone 145-2C11, #100325, Biolegend), and FITC anti-mouse CD4 (clone GK1.5, #100405, Biolegend). After fixation and permeabilization with a Cyto-Fast Fix/Perm Buffer Set (#426803, Biolegend), intracellular cytokine staining was performed with PE anti-mouse IFN (clone XMG1.2, #505807, Biolegend) and APC anti-mouse IL-4 (clone 11B11, #504105, Biolegend). Stained splenocytes were subjected to flow cytometry analysis using FACS Canto II with FACS Diva software (BD Biosciences, Inc., Franklin Lakes, NJ, USA).

### Statistical analysis

Statistical analysis was performed with a Mann–Whitney *U*-test or one-way analysis of variance (ANOVA), followed by Tukey’s multiple comparison test. *p*-values ≤ 0.05 were considered to indicate statistical significance.

### Reporting summary

Further information on research design is available in the Nature Research Reporting Summary linked to this article.

## Supplementary information

Supplementary Files Merged

REPORTING SUMMARY

Supplementary figure legends

## Data Availability

All data are presented in the main text or the supplementary materials and are available upon reasonable request from the corresponding author, Ryoma Nakao, by e-mail (ryoma73@niid.go.jp).

## References

[1] Kaparakis-Liaskos M, Ferrero RL (2015). Immune modulation by bacterial outer membrane vesicles. Nat. Rev. Immunol..

[2] Toyofuku M, Nomura N, Eberl L (2019). Types and origins of bacterial membrane vesicles. Nat. Rev. Microbiol..

[3] Schooling SR, Beveridge TJ (2006). Membrane vesicles: an overlooked component of the matrices of biofilms. J. Bacteriol..

[4] MacDonald IA, Kuehn MJ (2012). Offense and defense: microbial membrane vesicles play both ways. Res. Microbiol..

[5] Ladhani SN (2020). Vaccination of Infants with Meningococcal Group B Vaccine (4CMenB) in England. N. Engl. J. Med..

[6] Santana-Mederos D (2022). A COVID-19 vaccine candidate composed of the SARS-CoV-2 RBD dimer and *Neisseria meningitidis* outer membrane vesicles. RSC Chem. Biol.

[7] Micoli F (2018). Comparative immunogenicity and efficacy of equivalent outer membrane vesicle and glycoconjugate vaccines against nontyphoidal Salmonella. Proc. Natl Acad. Sci. USA.

[8] Irene C (2019). Bacterial outer membrane vesicles engineered with lipidated antigens as a platform for *Staphylococcus aureus* vaccine. Proc. Natl Acad. Sci. USA.

[9] Stevenson TC (2018). Immunization with outer membrane vesicles displaying conserved surface polysaccharide antigen elicits broadly antimicrobial antibodies. Proc. Natl Acad. Sci. USA.

[10] Price NL (2016). Glycoengineered Outer Membrane Vesicles: A Novel Platform for Bacterial Vaccines. Sci. Rep..

[11] Kruis W (2004). Maintaining remission of ulcerative colitis with the probiotic *Escherichia coli* Nissle 1917 is as effective as with standard mesalazine. Gut.

[12] Rembacken BJ, Snelling AM, Hawkey PM, Chalmers DM, Axon AT (1999). Non-pathogenic *Escherichia coli* versus mesalazine for the treatment of ulcerative colitis: a randomised trial. Lancet.

[13] Sturm A (2005). *Escherichia coli* Nissle 1917 distinctively modulates T-cell cycling and expansion via toll-like receptor 2 signaling. Infect. Immun..

[14] Canas MA, Fabrega MJ, Gimenez R, Badia J, Baldoma L (2018). Outer membrane vesicles from probiotic and commensal *Escherichia coli* activate NOD1-mediated immune responses in intestinal epithelial cells. Front. Microbiol..

[15] Rosenthal JA (2014). Mechanistic insight into the TH1-biased immune response to recombinant subunit vaccines delivered by probiotic bacteria-derived outer membrane vesicles. PLoS ONE.

[16] Hirayama S, Nakao R (2020). Glycine significantly enhances bacterial membrane vesicle production: a powerful approach for isolation of LPS-reduced membrane vesicles of probiotic *Escherichia coli*. Microb. Biotechnol.

[17] Hirayama S, Nakao R (2022). Glycine induction method: effective production of immunoactive bacterial membrane vesicles with low endotoxin content. Methods Mol. Biol..

[18] Bentley SD (2006). Genetic analysis of the capsular biosynthetic locus from all 90 pneumococcal serotypes. PLoS Genet..

[19] Bentley SD, Lo SW (2021). Global genomic pathogen surveillance to inform vaccine strategies: a decade-long expedition in pneumococcal genomics. Genome Med..

[20] Morimura A, Hamaguchi S, Akeda Y, Tomono K (2021). Mechanisms underlying pneumococcal transmission and factors influencing host-pneumococcus interaction: A review. Front. Cell. Infect. Microbiol..

[21] Kobayashi M (2022). Use of 15-valent pneumococcal conjugate vaccine and 20-valent pneumococcal conjugate vaccine among US adults: updated recommendations of the Advisory Committee on Immunization Practices-United States, 2022. MMWR Morb. Mortality Wkly Rep.

[22] Bahrs C (2022). A longitudinal analysis of pneumococcal vaccine serotypes in pneumonia patients in Germany. European Respir. J.

[23] Murthy N (2022). Advisory committee on immunization practices recommended immunization schedule for adultsaged 19 years or older - United States, 2022. MMWR Morb Mortal Wkly Rep.

[24] Rappuoli, R. Glycoconjugate vaccines: principles and mechanisms. *Sci. Transl. Med*. **10**, 10.1126/scitranslmed.aat4615 (2018). 10.1126/scitranslmed.aat4615 30158151

[25] Chen C (2019). Effect and cost-effectiveness of pneumococcal conjugate vaccination: a global modelling analysis. Lancet Glob. Health.

[26] Grozdanov L (2002). A single nucleotide exchange in the wzy gene is responsible for the semirough O6 lipopolysaccharide phenotype and serum sensitivity of *Escherichia coli* strain Nissle 1917. J. Bacteriol..

[27] Nissle A (1916). Über die Grundlagen einer neuen ursächlichen Bekämpfung der pathologischen Darmflora. Dtsch. Med. Wochenschr..

[28] Tetsutani K, Ishii KJ (2012). Adjuvants in influenza vaccines. Vaccine.

[29] Chen WH (2009). Vaccination in the elderly: an immunological perspective. Trends Immunol..

[30] Kumar R, Burns EA (2008). Age-related decline in immunity: implications for vaccine responsiveness. Expert Rev. Vaccines.

[31] Lin AY (2013). High-density sub-100-nm peptide-gold nanoparticle complexes improve vaccine presentation by dendritic cells in vitro. Nanoscale Res. Lett..

[32] Bandyopadhyay A, Fine RL, Demento S, Bockenstedt LK, Fahmy TM (2011). The impact of nanoparticle ligand density on dendritic-cell targeted vaccines. Biomaterials.

[33] Vu MN, Kelly HG, Kent SJ, Wheatley AK (2021). Current and future nanoparticle vaccines for COVID-19. EBioMedicine.

[34] Weaver GC (2016). In vitro reconstitution of B cell receptor-antigen interactions to evaluate potential vaccine candidates. Nat. Protoc..

[35] Senapati S (2021). Self-assembling synthetic nanoadjuvant scaffolds cross-link B cell receptors and represent new platform technology for therapeutic antibody production. Sci. Adv..

[36] Little SR (2012). Reorienting our view of particle-based adjuvants for subunit vaccines. Proc. Natl Acad. Sci. USA.

[37] Reddy ST (2007). Exploiting lymphatic transport and complement activation in nanoparticle vaccines. Nat. Biotechnol..

[38] Plotkin S, Robinson JM, Cunningham G, Iqbal R, Larsen S (2017). The complexity and cost of vaccine manufacturing-an overview. Vaccine.

[39] Nikaido H (2003). Molecular basis of bacterial outer membrane permeability revisited. Microbiol Mol. Biol. Rev..

[40] Raetz CR, Whitfield C (2002). Lipopolysaccharide endotoxins. Annu. Rev. Biochem.

[41] Sun J, Rutherford ST, Silhavy TJ, Huang KC (2022). Physical properties of the bacterial outer membrane. Nat. Rev. Microbiol..

[42] Okuda S, Sherman DJ, Silhavy TJ, Ruiz N, Kahne D (2016). Lipopolysaccharide transport and assembly at the outer membrane: the PEZ model. Nat. Rev. Microbiol..

[43] Ashraf KU (2022). Structural basis of lipopolysaccharide maturation by the O-antigen ligase. Nature.

[44] Han W (2012). Defining function of lipopolysaccharide O-antigen ligase WaaL using chemoenzymatically synthesized substrates. J. Biol. Chem..

[45] Wang RF, Kushner SR (1991). Construction of versatile low-copy-number vectors for cloning, sequencing and gene expression in *Escherichia coli*. Gene.

[46] Westphal O, Jann K (1965). Bacterial lipopolysaccharides. Extraction with phenol-water and further applications of the procedure. Methods Carbohydr. Chem..

[47] Kawahara K, Tsukano H, Watanabe H, Lindner B, Matsuura M (2002). Modification of the structure and activity of lipid A in Yersinia pestis lipopolysaccharide by growth temperature. Infect. Immun..

[48] Bradford MM (1976). A rapid and sensitive method for the quantitation of microgram quantities of protein utilizing the principle of protein-dye binding. Anal. Biochem.

[49] Pavlović Ž, Risović D, Novaković D (2012). Comparative study of direct and indirect image‐based profilometry in characterization of surface roughness. Surf. Interface Anal..

[50] Orskov I, Orskov F, Jann B, Jann K (1977). Serology, chemistry, and genetics of O and K antigens of *Escherichia coli*. Bacteriol. Rev..

[51] Wai SN (2003). Characterization of dominantly negative mutant ClyA cytotoxin proteins in *Escherichia coli*. J. Bacteriol..

[52] Ramstedt M, Nakao R, Wai SN, Uhlin BE, Boily J-F (2011). Monitoring surface chemical changes in the bacterial cell wall: multivariate analysis of cryo-x-ray photoelectron spectroscopy data. J. Biol. Chem..

[53] Shchukarev A, Gojkovic Z, Funk C, Ramstedt M (2020). Cryo-XPS analysis reveals surface composition of microalgae. Appl. Surf. Sci..

[54] Nakao R, Ramstedt M, Wai SN, Uhlin BE (2012). Enhanced biofilm formation by *Escherichia coli* LPS mutants defective in Hep biosynthesis. PLoS ONE.

[55] Nakao R, Myint SL, Wai SN, Uhlin BE (2018). Enhanced biofilm formation and membrane vesicle release by *Escherichia coli* expressing a commonly occurring plasmid gene, kil. Front. Microbiol..

