# Supplementary material for: A highly immunogenic vaccine platform against encapsulated pathogens using chimeric probiotic Escherichia coli membrane vesicles
Source: NPJ Vaccines. 2022 Nov 26;7:153. doi: 10.1038/s41541-022-00572-z (PMC9701205; doi:10.1038/s41541-022-00572-z)
Supplement: Supplementary file 1 — Supplementary Files Merged [file 41541_2022_572_MOESM1_ESM.pdf]

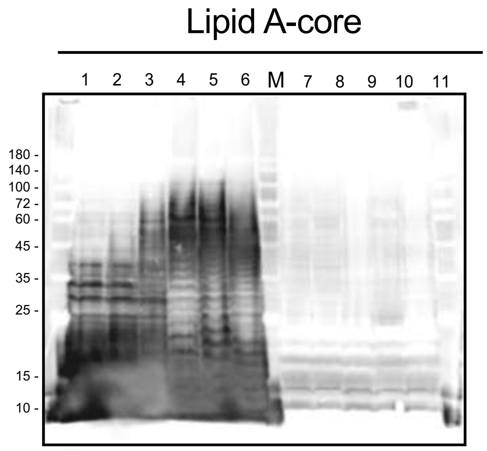


1. EcNΔ*flhD*/pWSK129 +IPTG
2. EcNΔ*flhD*/pNLP80 w/o IPTG
3. EcNΔ*flh*D/pNLP80 +IPTG
4. UPEC 536 (O6)
5. UPEC J96 (O4)
6. EHEC Sakai (O157)
7. *S. pneumoniae* ATCC700676, serotype 14
8. *S. pneumoniae* KSP1094, serotype 14
9. *S. pneumoniae* D39, serotype 2
10. *S. pneumoniae* M4, serotype 3
11. *S. pneumoniae* TIGR4, serotype 4

Supplementary Figure 1. Nakao et al.

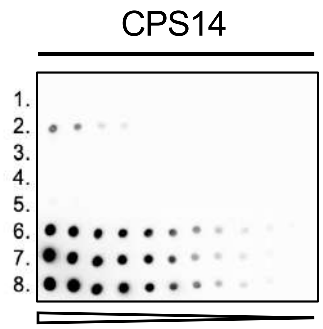


1. EcNΔ*flhD*/pWSK129 +IPTG 0.01mM
2. EcNΔ*flhD*/pNLP80 +IPTG 0.01mM
3. UPEC 536 (O6)
4. UPEC J96 (O4)
5. EHEC Sakai (O157)
6. *S. pneumoniae* ATCC 700676 (CPS14)
7. CPS14 standard-1
8. CPS14 standard-2

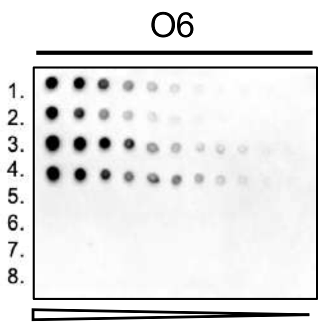


1. EcNΔ*flhD*/pWSK129 +IPTG 0.01mM
2. EcNΔ*flhD*/pNLP80 +IPTG 0.01mM
3. *E. coli* O6 reference strain (O6)
4. UPEC strain 536 (O6)
5. UPEC strain J96 (O4)
6. EHEC srain Sakai (O157)
7. *S. pneumoniae* ATCC 700676 (serotype 14)
8. *S. pneumoniae* KSP1094 (serotype 14)

Supplementary Figure 2. Nakao et al.

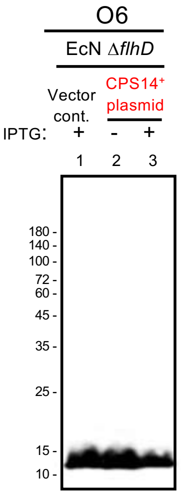


1. EcNΔ*flhD*/pWSK129 +IPTG
2. EcNΔ*flhD*/pNLP80 w/o IPTG
3. EcNΔ*flhD*/pNLP80 +IPTG

Supplementary Figure 3. Nakao et al.

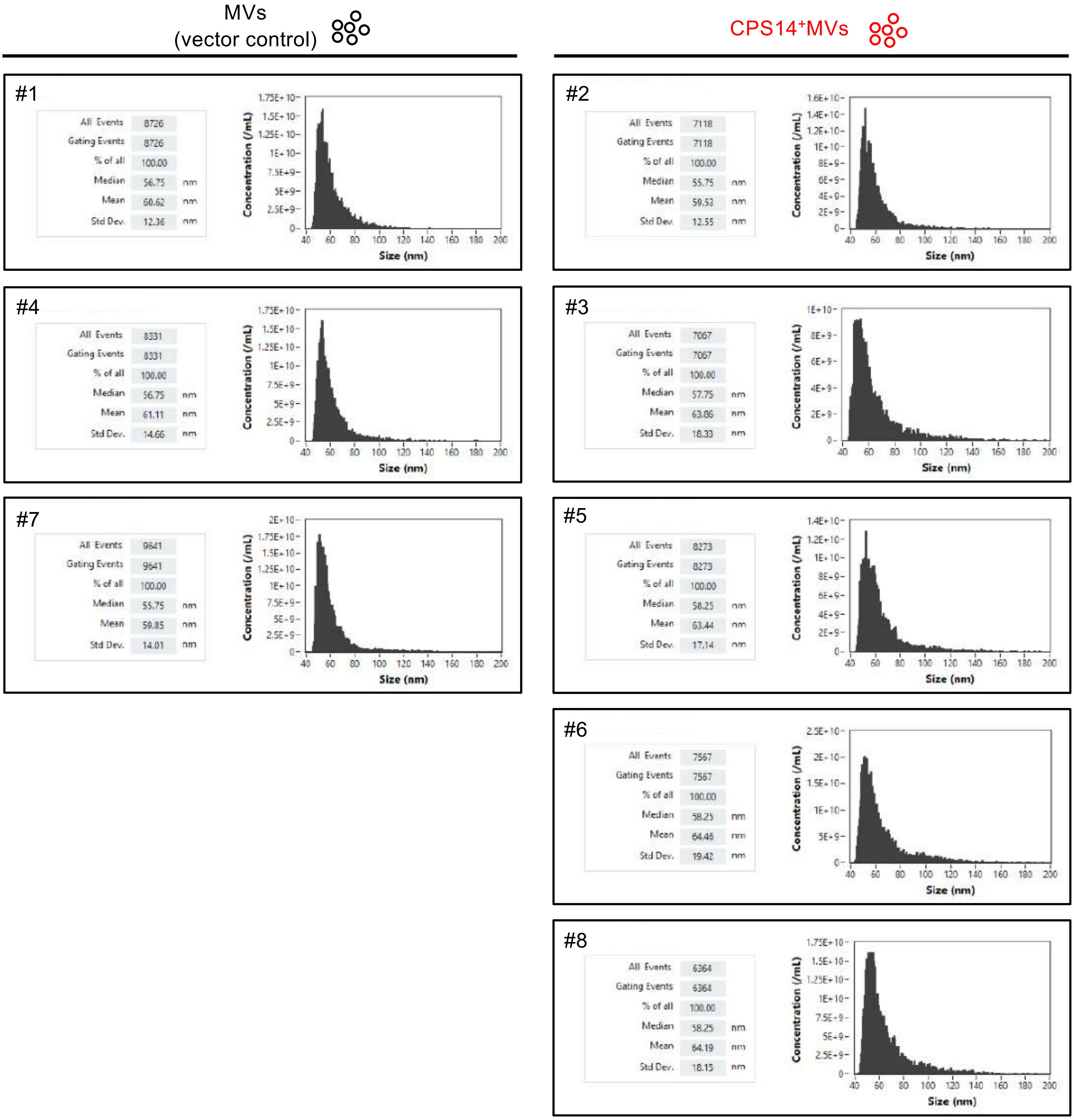


Supplementary Figure 4. Nakao et al.

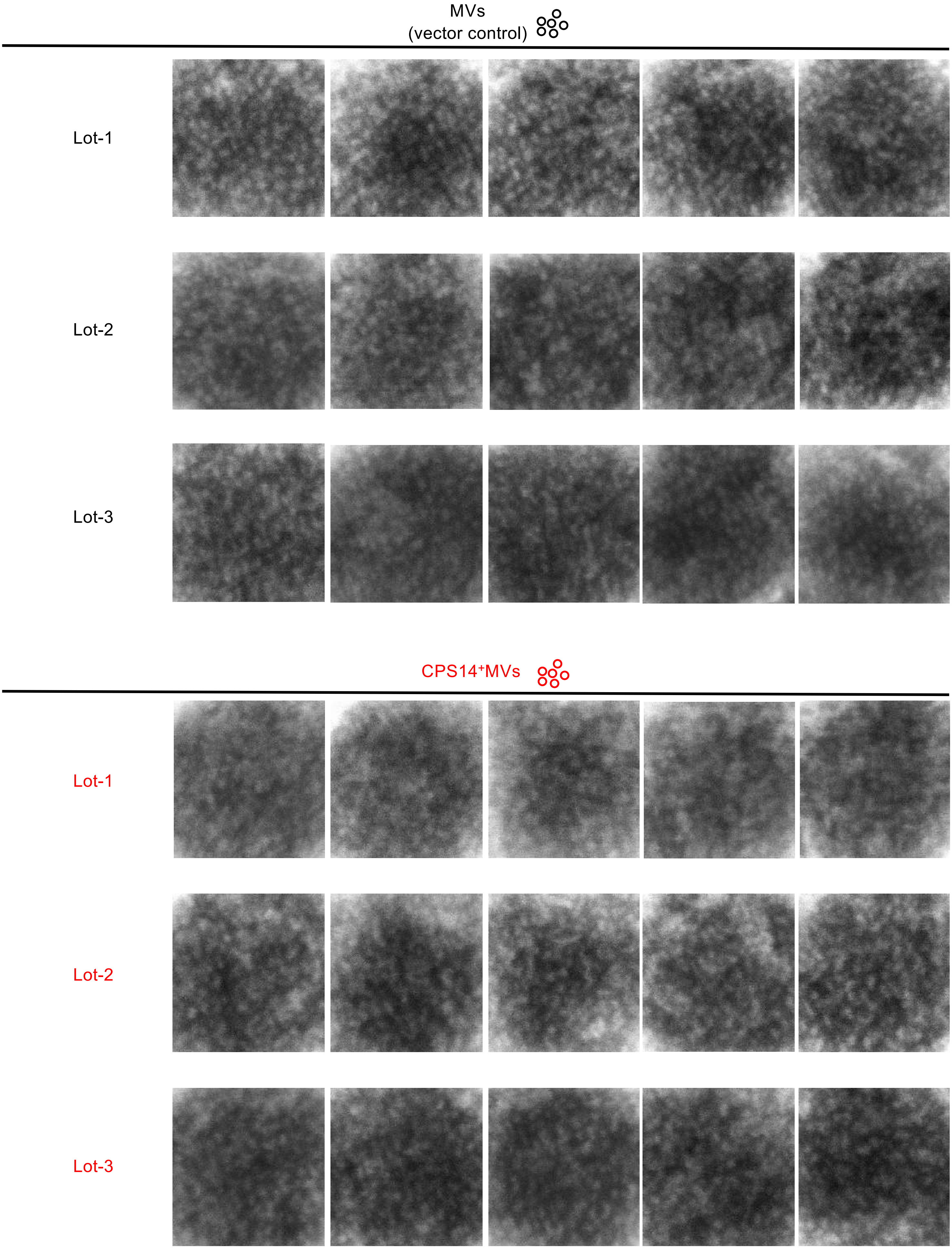


Supplementary Figure 5. Nakao et al.

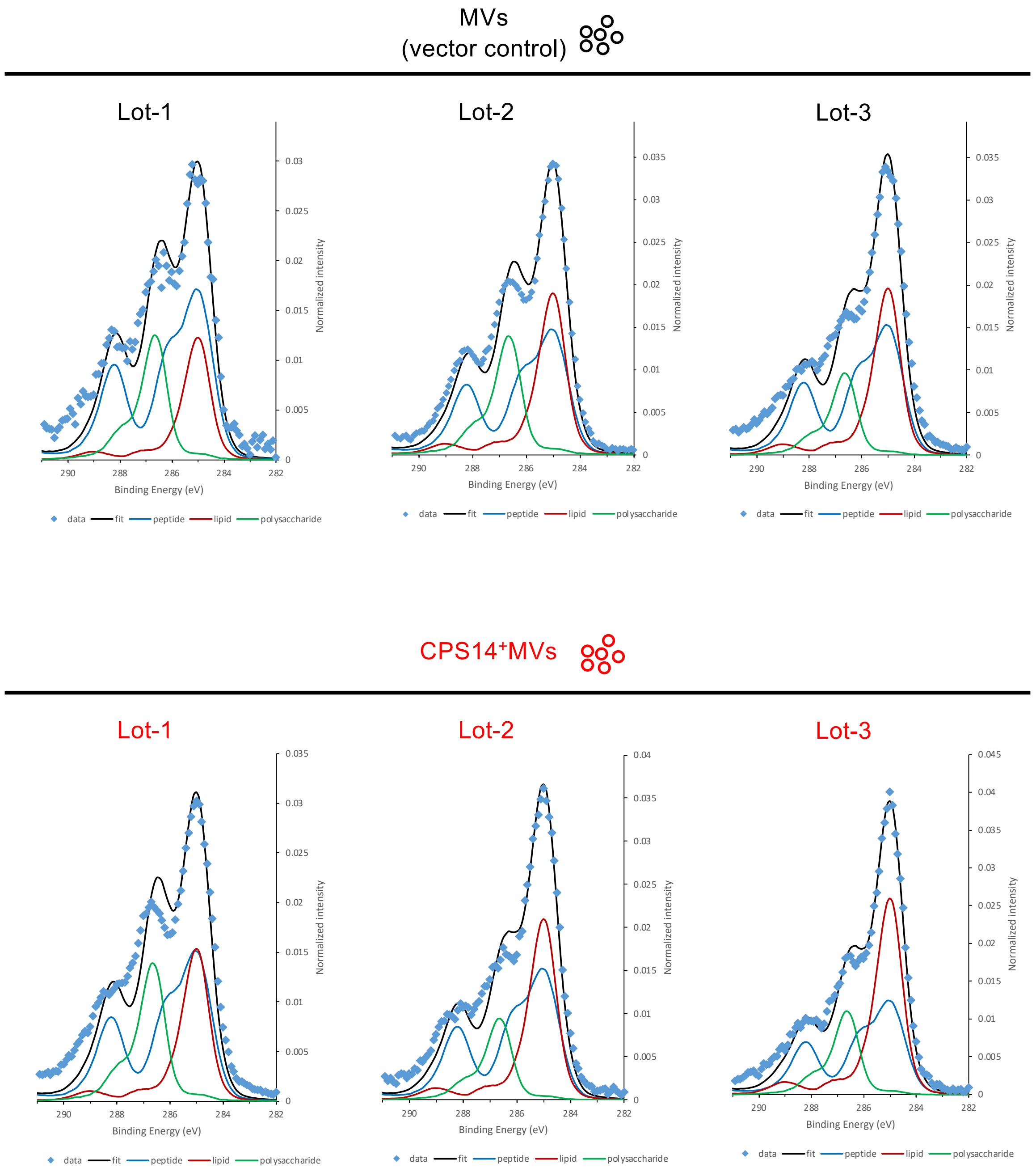


Supplementary Figure 6. Nakao et al.

## (A) Timeline of immunization, different doses of vaccines

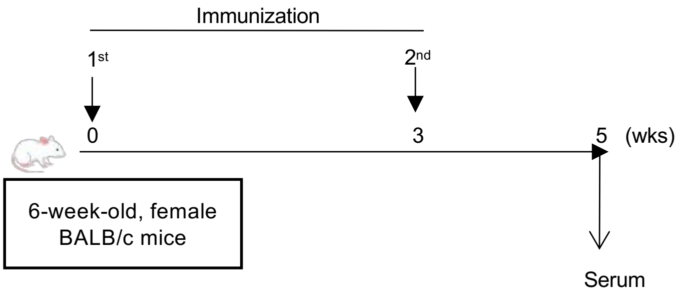


PPSV23_PBS and 3 different doses, 90 μL/shot
Mice (n=2) were administered with PBS, 1/9 diluted [containing 0.5 μg CPS14], 1/3 diluted [1.5 μg], and undiluted PPSV23 [4.5 μg].

PCV13_PBS and 3 different doses, 90 μL/shot
Mice (n=2) were administered with PBS, 1/9 diluted [containing 0.043 μg CPS14], 1/3 diluted [0.13 μg], and undiluted PCV13 [0.39 μg].

$CPS14^{+}MVs$_PBS and 3 different doses, 90 μL/shot
Mice (n=2) were administered with PBS, 1/9 diluted [containing 0.0014 μg CPS14], 1/3 diluted [0.0044 μg], and undiluted $CPS14^{+}MVs$ [0.013 μg].

## (B) Humoral immune responses in serum after vaccination with different doses

### PPSV23

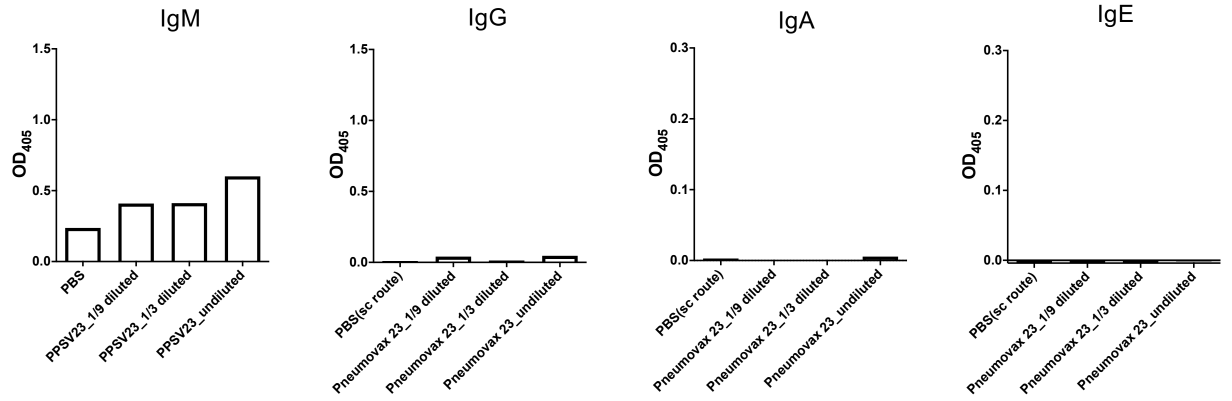


### PCV13

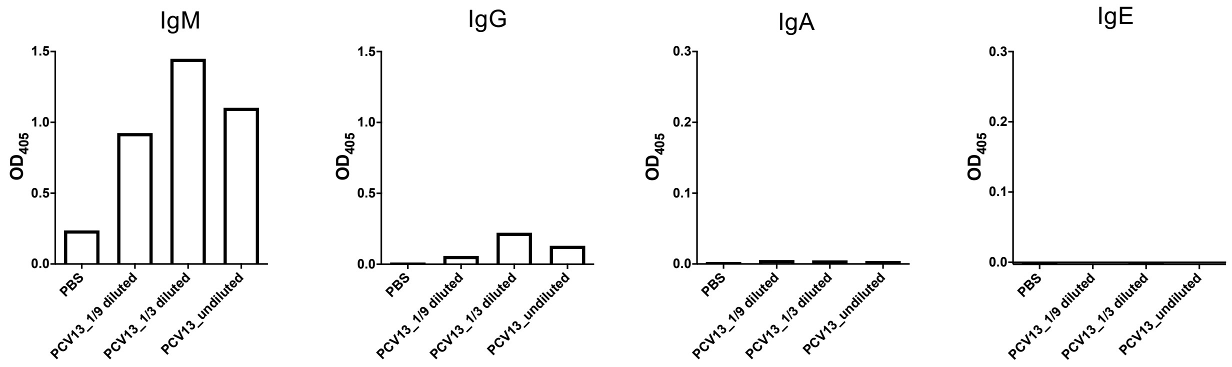


### $CPS14^{+}$ MVs

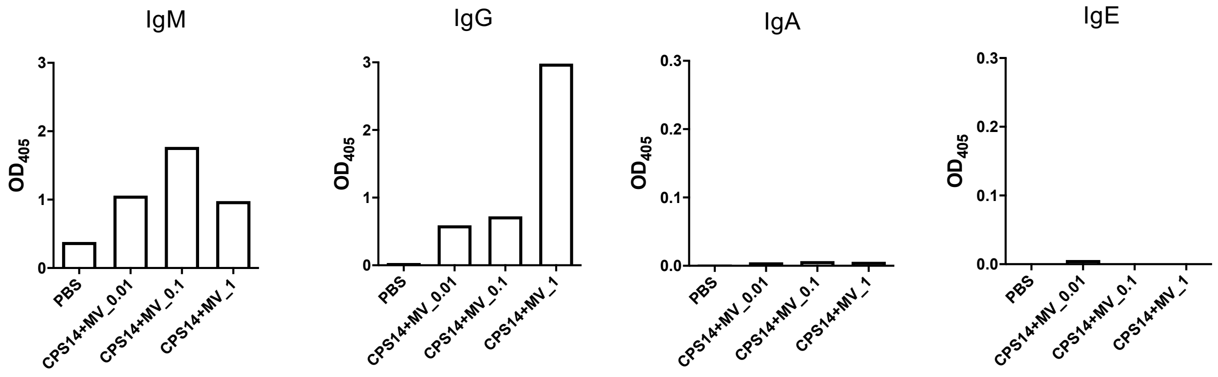


Supplementary Figure 7. Nakao et al.

(C) Vaccination with PPSV23, PCV13, and CPS14$^{+}$MVs at same CPS14 doses

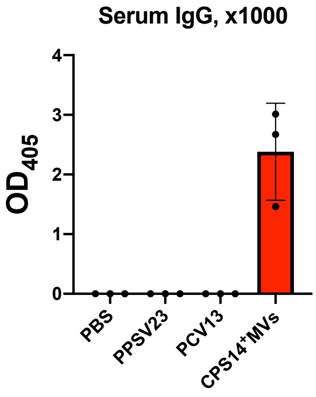


Supplementary Figure 7. Nakao et al.

(A) SDS resistance

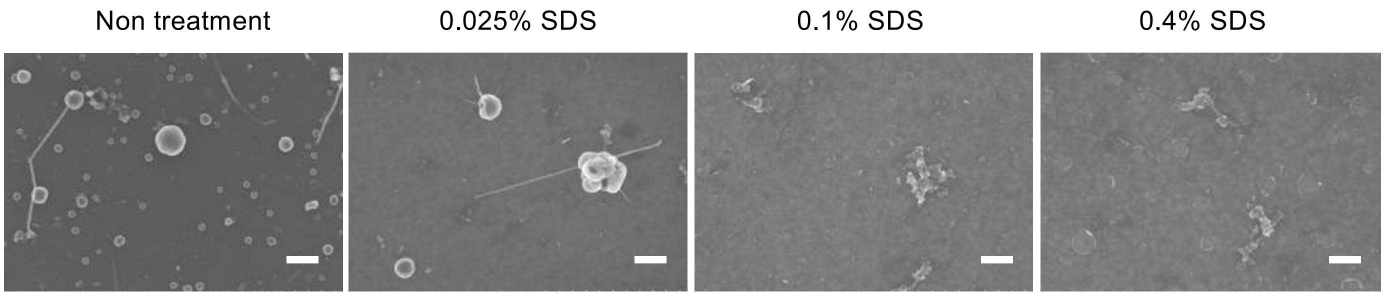


(B) Timeline of immunization, using CPS14⁺MVs treated with formalin or detergent

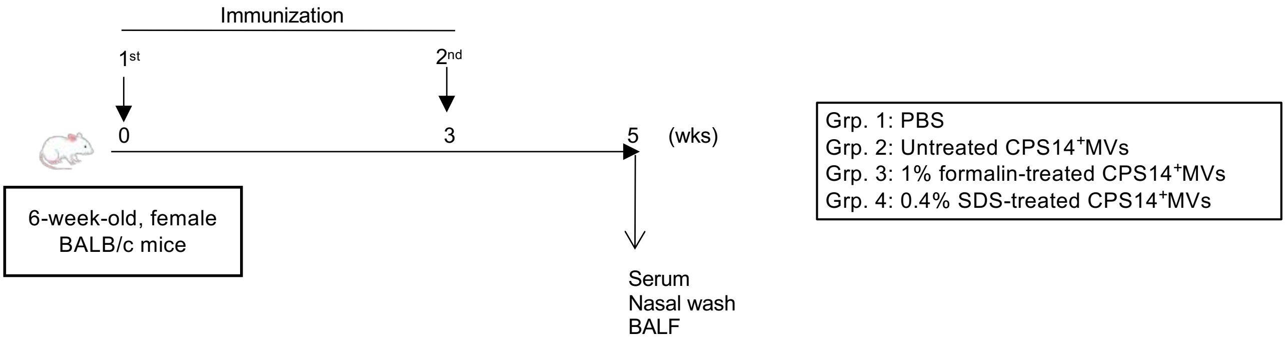


(C) Humoral immune responses against CPS14: treatment with formalin or SDS

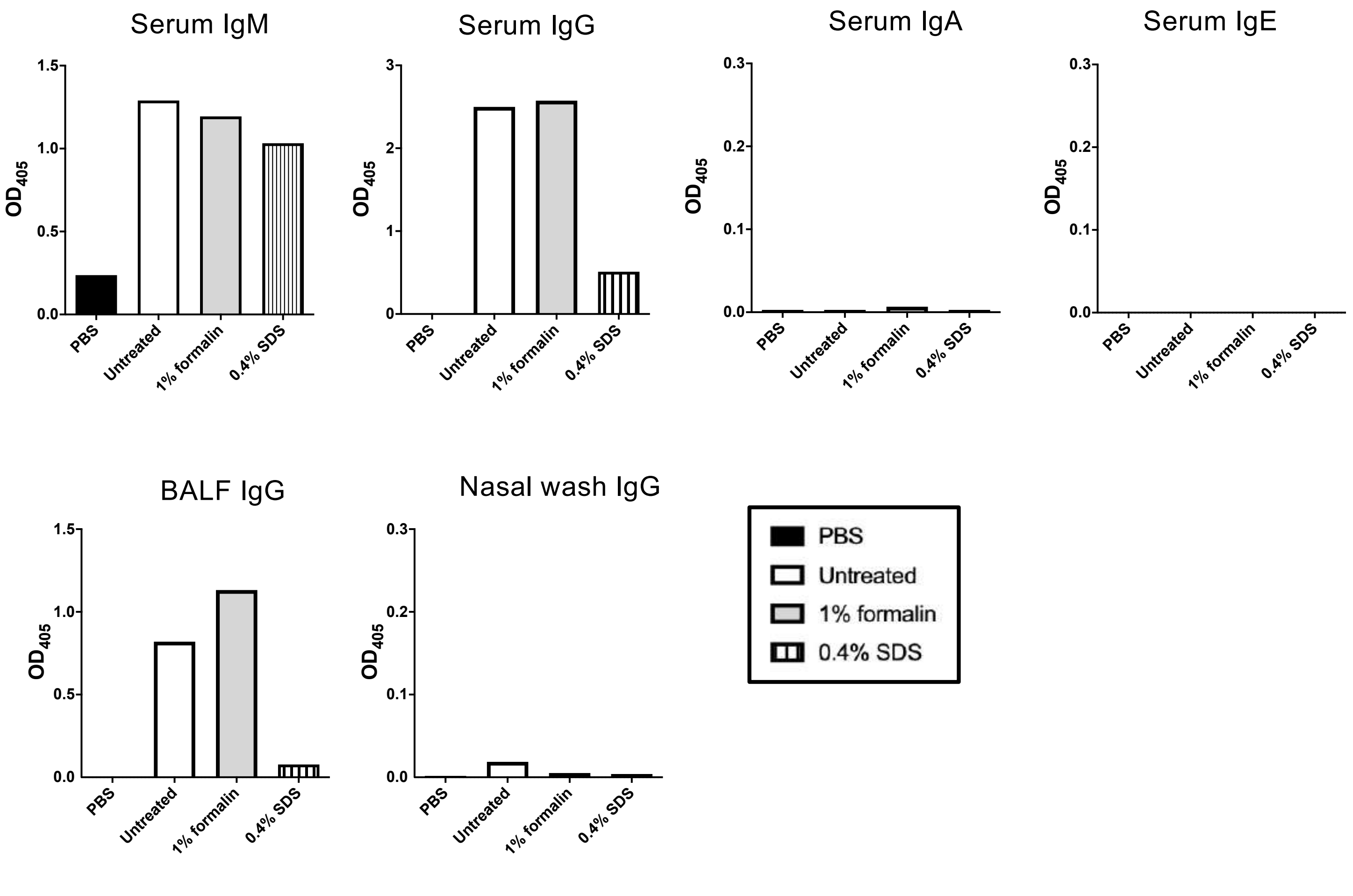


Supplementary Figure 8. Nakao et al.

(D) Timeline of immunization, using CPS14⁺MVs with or without heat treatment

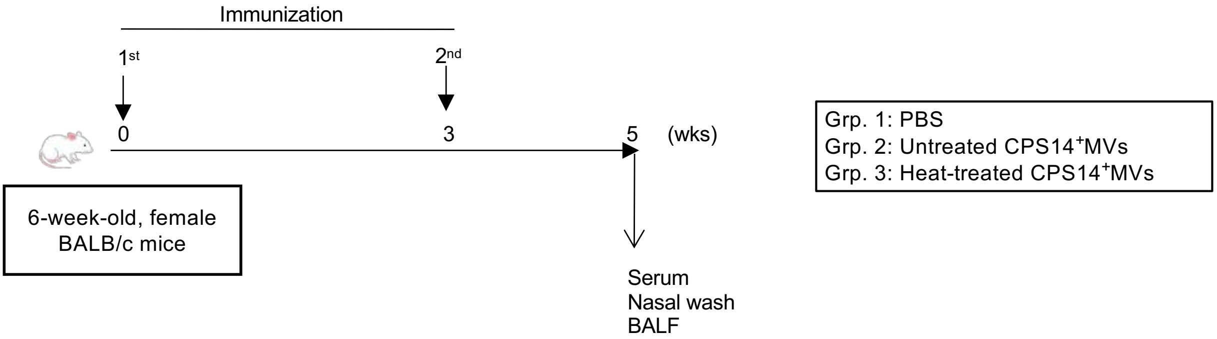


(E) Humoral immune responses against CPS14: Heat treatment

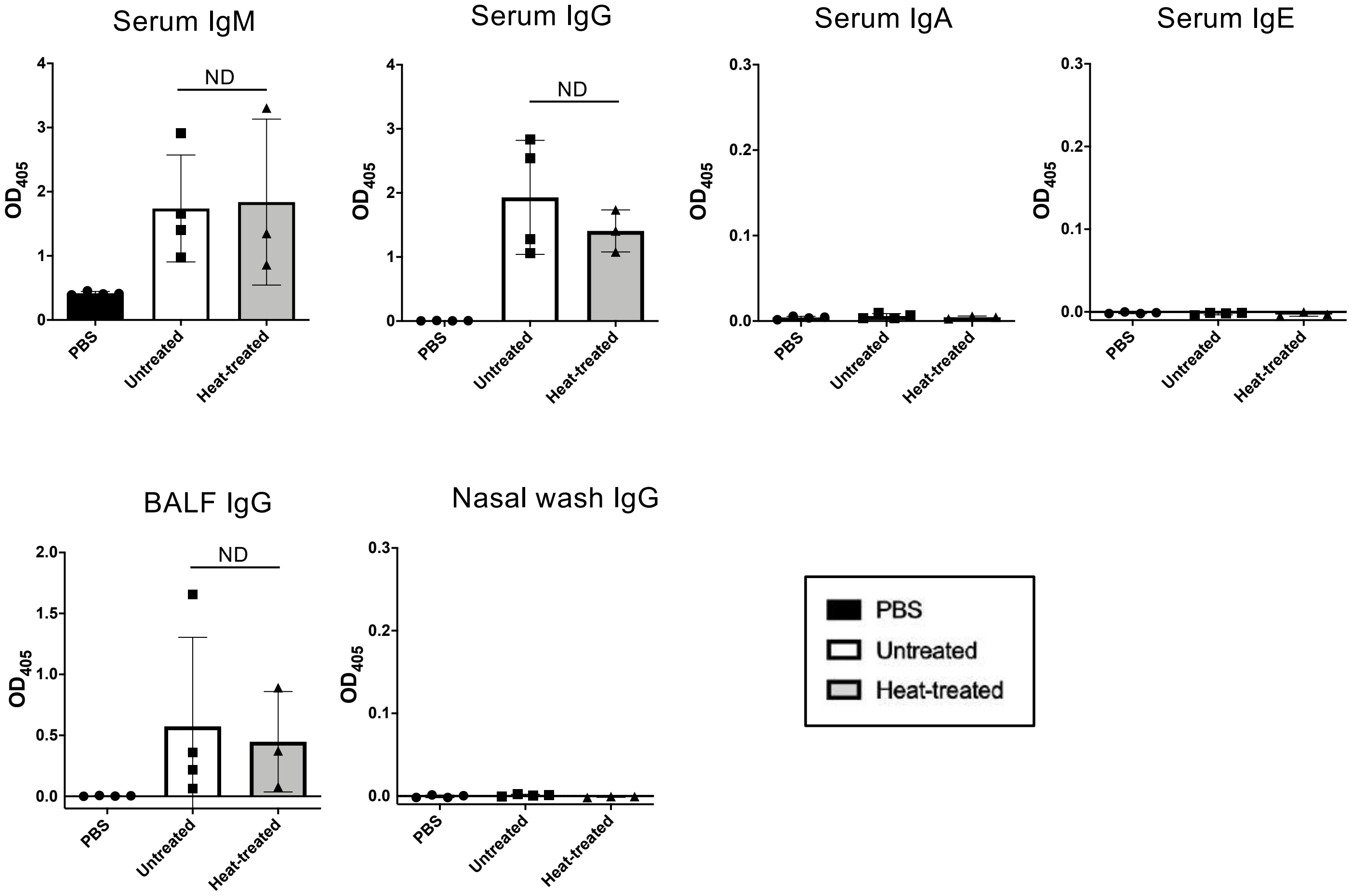


Supplementary Figure 8. Nakao et al.

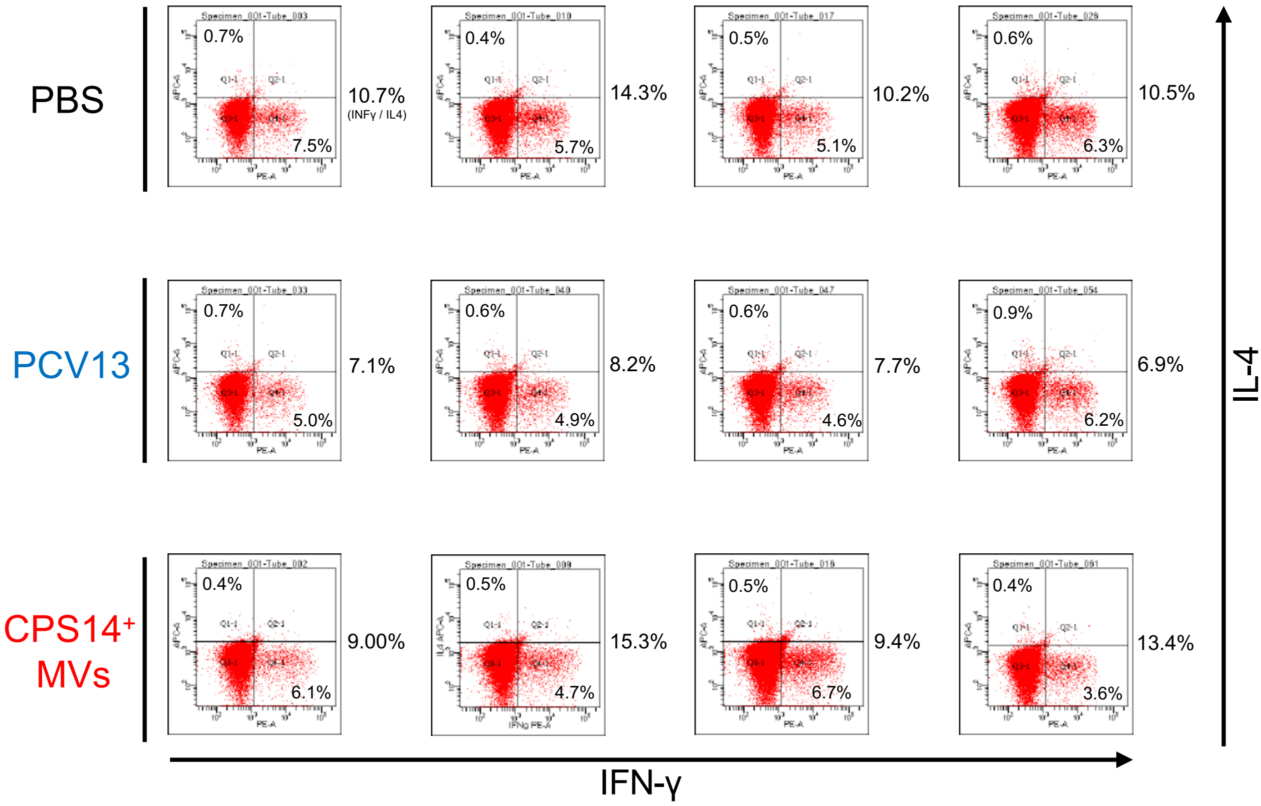


Supplementary Figure 9. Nakao et al.

(A) Secondary electron images

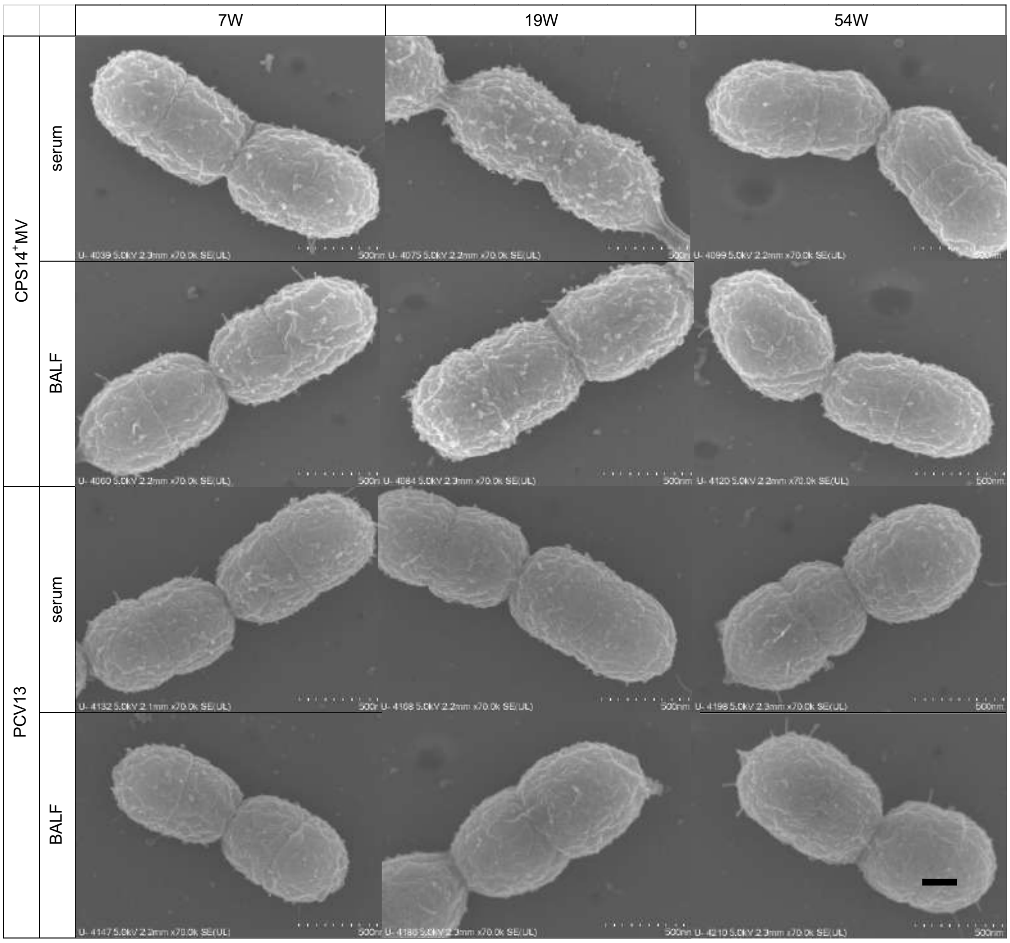


(B) Refractory electron images

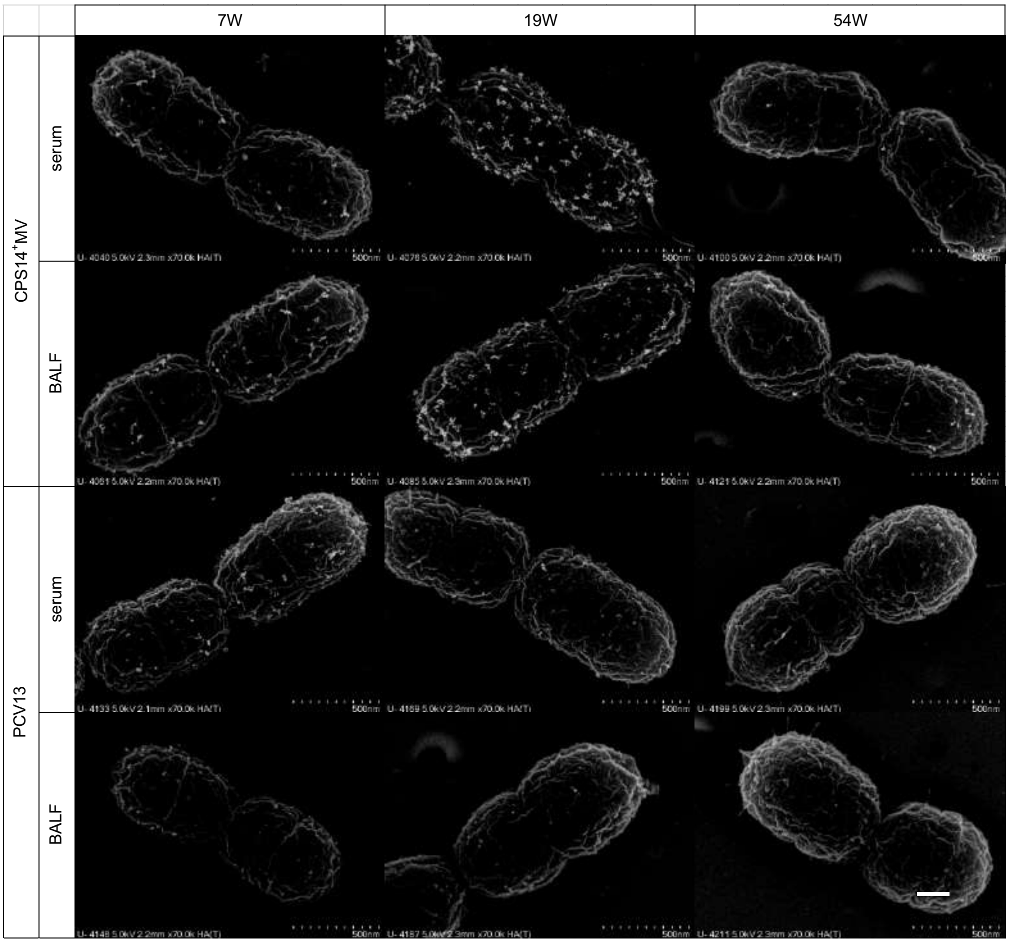


Supplementary Figure 10. Nakao et al.
