# Supplementary material for: A highly immunogenic vaccine platform against encapsulated pathogens using chimeric probiotic Escherichia coli membrane vesicles
Source: NPJ Vaccines. 2022 Nov 26;7:153. doi: 10.1038/s41541-022-00572-z (PMC9701205; doi:10.1038/s41541-022-00572-z)
Supplement: Supplementary file 3 — Supplementary figure legends [file 41541_2022_572_MOESM3_ESM.docx]

**Supplementary figure legends**

**Title:**

A highly immunogenic vaccine platform against encapsulated pathogens using chimeric probiotic *Escherichia coli* membrane vesicles

**Authors:**

Ryoma Nakao^a*^, Hirotaka Kobayashi^b^, Yusuke Iwabuchi^a,c^, Kazuyoshi Kawahara^d^, Satoru Hirayama^a,e^, Madeleine Ramstedt^f^, Yuki Sasaki^g^, Michiyo Kataoka^b^, Yukihiro Akeda^a^, Makoto Ohnishi^h^

**Affiliations:**

^a^ Department of Bacteriology I, National Institute of Infectious Diseases, 1-23-1, Toyama, Shinjuku-ku, Tokyo 162-8640, Japan

^b^ Department of Pathology, National Institute of Infectious Diseases, 1-23-1, Toyama, Shinjuku-ku, Tokyo 162-8640, Japan

^c^ Department of Pediatric Dentistry/Special Needs Dentistry, Graduate School of Medical and Dental Sciences, Tokyo Medical and Dental University, 1-5-45, Yushima, Bunkyo-ku, Tokyo 113-8519, Japan.

^d^ College of Science and Engineering, Kanto Gakuin University, 1-50-1, Mutsuura-higashi, Kanazawa-ku, Yokohama, Kanagawa 236-8501, Japan

^e^ Division of Microbiology and Infectious Diseases, Niigata University Graduate School of Medical and Dental Sciences, 2-5274, Gakkocho-dori, Chuo-ku, Niigata 951-8514, Japan

^f^ Department of Chemistry, Umeå Centre for Microbial Research (UCMR), Umeå University, SE-90187 Umeå, Sweden

^g^ Nanostructures Research Laboratory, Japan Fine Ceramics Center, 2-4-1 Mutsuno, Atsuta-ku, Nagoya 456-8587, Japan

^h^ National Institute of Infectious Diseases, 1-23-1, Toyama, Shinjuku-ku, Tokyo 162-8640, Japan

*Corresponding author:

E-mail: [ryoma73@nih.go.jp](mailto:ryoma73@nih.go.jp)

**Supplementary Figure 1. Lipid A-core oligosaccharide-probed western blot analysis on whole cells from a series of *E. coli* and *S. pneumoniae* organisms**

The bacterial strains used in this experiment are shown with their lane numbers in the lower right. Whole cells of all examined strains of *E. coli* and *S. pneumoniae* were standardized at an OD_600_ of 4.0 or 8.0, respectively. Twenty microliters of each sample was applied to 12.5% polyacrylamide SDS-PAGE, electro-transferred onto PVDF membrane, and probed with an anti-lipid A-core oligosaccharide antibody. Strong signals ranging from 40 to 80 kDa appeared in the EcNΔ*flhD* strain with exogenous CPS14 (Lane 3). The specificity of the anti-lipid A core oligosaccharide antibody was also confirmed by strong signals that appeared in all of the *E. coli* strains, and none of the *S. pneumoniae* strains.

**Supplementary Figure 2. Dot blot analysis for probing CPS14 and O6 antigens on *E. coli* and *S. pneumoniae***

Whole cells of all of the examined bacterial strains were standardized with PBS at an OD_600_ of 2.0. Purified CPS was standardized at 10 µg/mL. All samples were serially diluted 2-fold with PBS. Ten microliters of each sample was spotted onto a PVDF membrane, and dried. For detecting CPS14 and O6, the membranes were probed with an anti-CPS14 antibody or anti-O6 antibody. The bacterial strains used in these experiments are shown with their lane numbers at the bottom of each blot.

**Supplementary Figure 3. O6-probed western blot analysis on whole cells of EcN strains**

EcN cells standardized at an OD_600_ of 4.0 were subjected to O6-probed western blotting. The O6-specific signals were optimal for detecting long O-antigen, but not for semi-rough-type O6 LPS signals (Lanes 1, 2, 3; Fig. 2A). Therefore, the same samples were diluted 10-fold (OD_600_=0.4) and used for lipid A-core oligosaccharide-probed western blotting. The bacterial strains used in this experiment are shown with their lane numbers in the lower right.

**Supplementary Figure 4. Nano-flow cytometry of MVs (vector control) and CPS14^+^MVs**

The size distributions of MVs (vector control, n=3) and CPS14^+^MVs (n=5) were analyzed by using NanoFCM, and shown as a histogram. Data including median, mean and standard deviation for the MV samples are shown at the left in the column for each lot.

**Supplementary Figure 5. FE-SEM analysis: surface appearance of MVs at high magnification**

The surface appearances of MVs (vector control) and CPS14^+^MVs were compared by using FE-SEM. Three batches of both MVs and CPS14+ MVs were isolated in three independent experiments. Five MV particles of each batch that are more than 60 nm in diameter were randomly chosen. Shown are the cropped center area of each MV particle (25 nm x 25 nm). The cropped images were used for surface roughness analysis.

**Supplementary Figure 6. Cryo-XPS spectrum data of MVs**

C1s spectrum of MVs (vector control) and CPS14^+^MVs, as presented in Figure 3C in the article. Three batches of both MVs and CPS14^+^ MVs were isolated in three independent experiments. The spectra show the fitting of C 1s spectra using spectral components predicting the chemical content of lipid (red), polysaccharide (green) and peptide (protein + peptidoglycan, blue), as atomic percent. The raw data are represented by blue diamonds, the different components by colored lines, and the fit of the model to the data is represented by the black solid line.

**Supplementary Figure 7. Humoral immune responses after immunization with PPSV23, PCV13, and CPS14^+^ MVs: dose-response analyses**

(A) Timeline of immunization: different doses of vaccines, PPSV23, PCV13, and CPS14^+^MVs. Six-week-old, female BALB/c mice were subcutaneously immunized for twice at weeks 0 and 3, with different doses of PPSV23 (0.5, 1.5 and 4.5 µg of CPS14), PCV13 (0.04, 0.1, and 0.4 µg of CPS14), and CPS14^+^ MVs (0.0015, 0.0044 and 0.013 µg of CPS14). At 5 weeks, the serum samples were collected. The number of mice used in the preliminary experiments were two per group. (B) Humoral immune responses against CPS14. Serum IgM, IgG, IgA, and IgE were examined. In all ELISAs except serum IgG, samples were used at 1:100 times dilutions. In ELISA for serum IgG, the samples were used at 1:1,000 dilution. The results are expressed as OD_405_ (mean) after a 30-min incubation with AP substrate. (C) CPS14-specific Serum IgG responses after vaccination with PPSV23, PCV13, and CPS14^+^ MVs containing same CPS 14 dose (0.013 µg). The number of mice used were three per group. Serum samples were used at 1:1,000 dilution. The results are expressed as OD_405_ (mean ± SD) after a 45-min incubation with AP substrate.

**Supplementary Figure 8. Effect of MV structure integrity on humoral immune responses**

(A) Resistance to detergent. Susceptibility of CPS14^+^MVs to detergent was examined. CPS14^+^MVs samples were treated with SDS at different concentrations (none, 0.025%, 0.1%, and 0.4% SDS). Representative images were shown. Bars: 100 nm.

(B) Timeline of immunization: treatment with PBS alone, 1% formalin, and 0.4% SDS. Six-week-old, female BALB/c mice were subcutaneously immunized for twice at weeks 0 and 3, with PBS alone, CPS14^+^MVs untreated, and treated with 1% formalin, and 0.4% SDS. The number of mice used in the preliminary experiments were two per group.

(C) Humoral immune responses against CPS14 after treatment with formalin or SDS. Serum IgM, IgG, IgA, and IgE, as well as BALF and nasal wash IgG were examined. In all ELISAs except serum IgG, samples were used at 1:100 dilutions. In ELISA for serum IgG, the samples were used at 1:1,000 dilution. The results are expressed as OD_405_ (mean ± SD) after a 30-min incubation with AP substrate. (D) Timeline of immunization: heat treatment. Six-week-old, female BALB/c mice were subcutaneously immunized twice at weeks 0 and 3, with PBS alone (n=4), CPS14^+^MVs untreated (n=4), and treated with heat at 100 °C for 30 min (n=3).

(E) Humoral immune responses against CPS14 after treatment with heat. Serum IgM, IgG, IgA, and IgE, as well as BALF and nasal wash IgG were examined. For all ELISAs except serum IgG, samples were used at a dilution of 1:100. For serum IgG ELISA, the samples were used at a dilution of 1:1,000. Data are expressed as the mean ± SD. Statistical analysis was performed with one-way ANOVA, followed by Tukey’s multiple comparison test. ND: No statistically significant difference.

**Supplementary Figure 9. T cell responses after immunization with PBS PCV13, or CPS14^+^ MVs**

Mice were euthanized 4 weeks after the second immunization (Fig. 5E). Mouse splenocytes following treatment with a cell activation cocktail including brefeldin A for 6 hours were subjected to flow cytometry analysis. Shown are results of spleen CD4^+^T cells from mice subcutaneously immunized with PBS, PCV13, or CPS14 (n=4 per group). In each of the quadrant dot plots, IL-4^+^ and INF-γ^+^cell populations are shown in upper left and lower right columns, respectively. The ratio of IL-4^+^/INF-γ^+^ cells are also shown at the right of the columns.

**Supplementary Figure 10. FE-SEM analysis: counting gold particles on pneumococcal cells**

FE-SEM images of 100 cells were randomly captured for each group at 5 × 10^5^-fold magnification. Shown are representative the secondary electron images (A) and the refractory electron images (B) of *S. pneumoniae* (KSP1094, serotype-14, a clinical isolate from a patient with bronchitis and sepsis) after probing by different mouse serum and BALF samples which were further probed with 10-nm colloidal gold-labeled goat anti-mouse IgG (H+L) antibody. A rectangular area defined as 180 × 250 nm^2^ was cropped from the center of a pneumococcal cell. The number of immuno-gold particles on the surface per cell was counted for assessment of the immunoreactivity of the serum and BALF samples. Bars: 200 nm.
